# Supplementary material for: Permeation enhancer-induced membrane defects assist the oral absorption of peptide drugs
Source: Nat Commun. 2025 Oct 28;16:9512. doi: 10.1038/s41467-025-64891-0 (PMC12568990; doi:10.1038/s41467-025-64891-0)
Supplement: Supplementary file 1 — Supplementary Information [file 41467_2025_64891_MOESM1_ESM.pdf]

SUPPLEMENTARY INFORMATION

Colston, Faivre, and Schneebeli

SUPPLEMENTARY METHODS

I. General Experimental Methods and Materials

Heating and stirring was carried out with a Heidolph MR Hei-TEC 145 mm diameter magnetic stirring hotplate with a Pt1000 temperature sensor. All dynamic light scattering (DLS) spectra were recorded with a Zetasizer Advance Series — Ultra (Red) Instrument with a 10mW HeNe laser (633nm) with high sensitivity avalanche photodiode (APD) detector. The DLS data was analyzed with the Malvern Panalytical's ZS Xplorer (version 3.3.0.42) application software on a Windows host computer.

Unless otherwise noted, ^{1}H NMR spectra were recorded under automation using IconNMR with 24-position sample case at 298 K on a Bruker NEO500-2 (500 MHz) spectrometer, equipped with a 5 mm VT probe with BBFO configuration, two RF transmit/receive channels, Z-gradient hardware, variable temperature control with a BCU-II chiller unit, and a Linux host computer running TopSpin 4.3.0. The spectra were referenced to the residual solvent peak (chloroform-*d*: 7.26 ppm for ^{1}H-NMR) or to an internal TMS or DSS standard if available. Chemical shift values (δ) were recorded in parts per million (ppm).

Neutral 8-(2-hydroxybenzamindo)octanoic acid (SNAC-H, Cat. No. A941809) and salcaprozate sodium (SNAC, Cat. No. A224096) were purchased from Ambeed and used as received. Hexadecyltrimethylammonium bromide (CTAB, Cat. No. 227165000) was used as received from ThermoFisher Scientific. Semaglutide (sodium salt) was ordered from Cayman Chemical (Item No. 40170) and used as received. NMR grade tetramethyl silane (Cat. No.: AC138470250) and Chloroform-D (Cat. No.: DLM-29-0) were purchased from Thermo Scientific and Cambridge Isotope Laboratories, respectively, and used as received to make a 1% (v/v) TMS solution for the NMR titrations with TMS as the internal standard. NMR grade D_2O (Cat. No. DLM-4-100) and Sodium trimethylsilylpropanesulfonate (DSS, Cat. No. 178837-1G) were purchased from Cambridge Isotope Laboratories and Milipore Sigma, respectively, for ^{1}H and NOESY NMR experiments of CTAB and SNAC.

II. Molecular Dynamics Simulations

Software and Force Fields Used for the MD Simulations. All simulations were performed with a special fork of GROMACS 2021[1] available on GitHub (https://gitlab.com/gromacs-constantph/constantph, accessed July 18, 2024), which implements a scalable version of C*p*HMD in GROMACS. The CHARMM36[2] and the CHARMM general force field (CGenFF)[3] was used for the lipids, the semaglutide peptide, and the small molecules while all the water in the system was modeled with the TIP3P water-model.[4] For the standard, titratable residues (His-5, Glu-7, Asp-15, Glu-21, Lys-26, Glu-27, Arg-34, and Arg-36) of semaglutide, the optimized parameter set developed by the Hess and Groenhof group[5] was used. This parameter set contains[5] some reduced torsional barriers for torsions involving the titratable atom in order to improve the conformational sampling of the functional groups treated with the constant *p*H model. Analogously, to enhance the conformational sampling of the non-standard titratable carboxylic acid functional groups in semaglutide and in SNAC, the CG321-CG2O2-OG311-HGP1 torsion was reduced (revised parameters are shown in Table S1) which provided sufficient sampling as shown by the consistent λ-coordinate histograms (Supplementary Figs. 8–15).

Setup of the MD Simulations. Membranes for SNAC and fatty acid chain pulling simulations were generated using the CHARMM-GUI[6] bilayer builder (64 POPC lipids per leaflet). The SNAC-containing lipid bilayer was constructed using the PACKMOL software[7] (108 POPC lipids, 72 cholesterol molecules, and 50 SNACs per leaflet). An initial steepest descent minimization was performed for 5,000 steps followed by two steps of equilibration. The first equilibration step (*NVT* ensemble) was performed with a 2 fs time step and the v-rescale coupling method to maintain a temperature of 310.15 K with a time constant of 0.5 ps. The second equilibration step (*NPT* ensemble) was performed using a 2 fs time step using c-rescale to maintain pressure semi-isotropically at 1.0 bar with a compressibility constant of 4.5 x 10^{-5} bar^{-1}. The cutoff distance for short-ranged nonbonded interactions was set to 1.2 nm with electrostatic interactions calculated using the PME method,[8] and constraints were imposed on all simulations using the LINCS algorithm.[9] Pulling simulations were performed with simple distance increases between defined pulling groups in the z direction in which the center of mass for each group was determined from the previous step. Simulations performed with C*p*HMD methods included at least 5 buffer atoms per titratable site to balance the total charge of the system. For all umbrella sampling simulations

presented in the manuscript, the initial membrane system was equilibrated for 100 ns. WHAM analysis was carried out with the standard protocol implemented in GROMACS,[1] with a tolerance for convergence of 1.0 x 10^{-6}.

Validation of Constant pH Parameters. Systems for parameterization were generated as outlined from the *phbuilder*[10] tutorial. Atomic charges and forcefield topologies for parameterized molecules were generated using the CHARMM-GUI[6] web-based tool. Minor changes were applied to the forcefield to improve sampling of carboxylic acid ionizable groups detailed above. SNAC was parameterized fully while the 26th residue for semaglutide was split into butyric acid and N^2-Acetyl-*N*-methylglutamine (Supplementary Fig. 21–24). To generate approximate parameters for the C*p*HMD simulations, these molecules were then placed in a 4.5 nm^3 box and solvated using GROMACS 2021. Topology files, initial protonation states of titratable residues, system neutralization, and parameter files were all generated/performed using *phbuilder*. Systems were neutralized to include 0.15 M NaCl and 10 buffer molecules per titratable residue. Next, initial $\partial V/\partial \lambda$ C*p*HMD coefficients for each titratable site were generated by performing 13 different 100 ns simulations. For each simulation, the molecule was restrained to the center of the box and a different starting λ value (ranging from –0.1 to 1.1) was used. The resulting λ values were fit to a 7th order polynomial (Supplementary Figs. 8–15) using the provided script on the *phbuilder* GitLab.[11] Finally, these parameters were validated by performing 10 different 100 ns unrestrained simulations with the *p*H of simulation equal to the pK_a and the energy barrier bewteen λ states set to zero. Appropriately parameterized groups show an equal distribution of λ values consistently across all validation replicas. No further refinement was required for these systems demonstrating that the protonation free energy for the titratable residue is being corrected appropriately.

SUPPLEMENTARY FIGURES

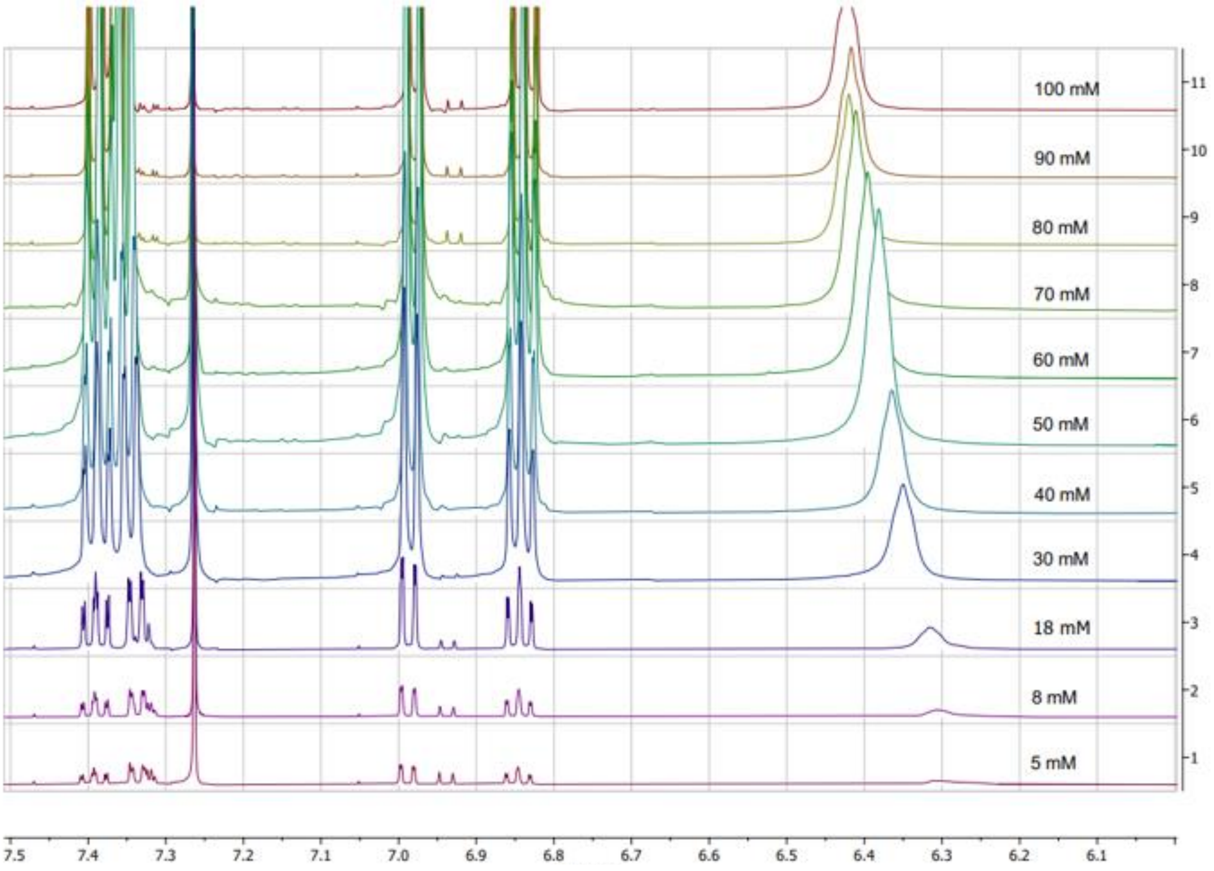

Supplementary Fig. 1 | Concentration-dependent ^{1}H NMR Resonances of Protonated SNAC in a $CDCl_3$ as a Model of the Membrane Interior. The stacked ^{1}H NMR spectra (500 MHz, $CDCl_3$, 298 K) are referenced to the TMS internal standard and focused on the region of the amide-NH resonance. Each spectrum is labeled with its corresponding SNAC concentration. The amide resonance (δ ≈ 6.35 ppm) shifts downfield due to hydrogen bonding interactions.

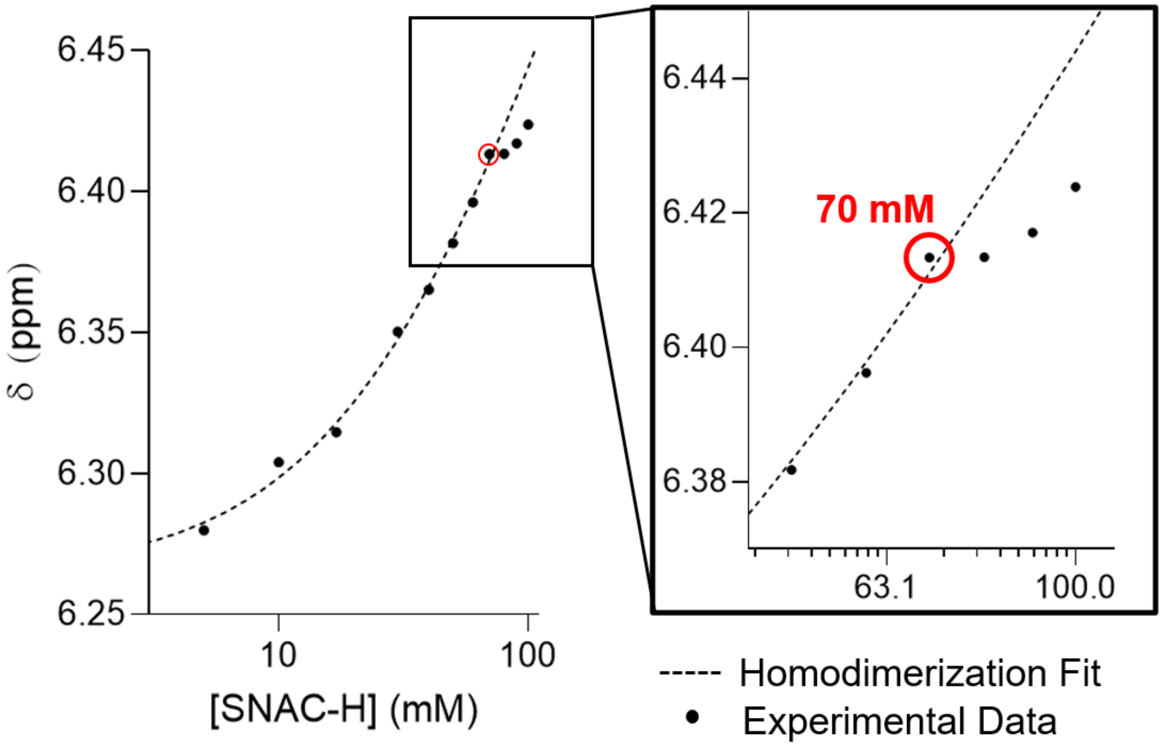

Supplementary Fig. 2 | ^{1}H NMR Titration (500 MHz, $CDCl_3$, 298 K) of SNAC Monitoring the Chemical Shift of the Amide Peak. The shift in the amide resonance was modeled using a 1:1 homodimerization binding model with Dynafit (version 4.11.110). Source data are provided as a Source Data file.

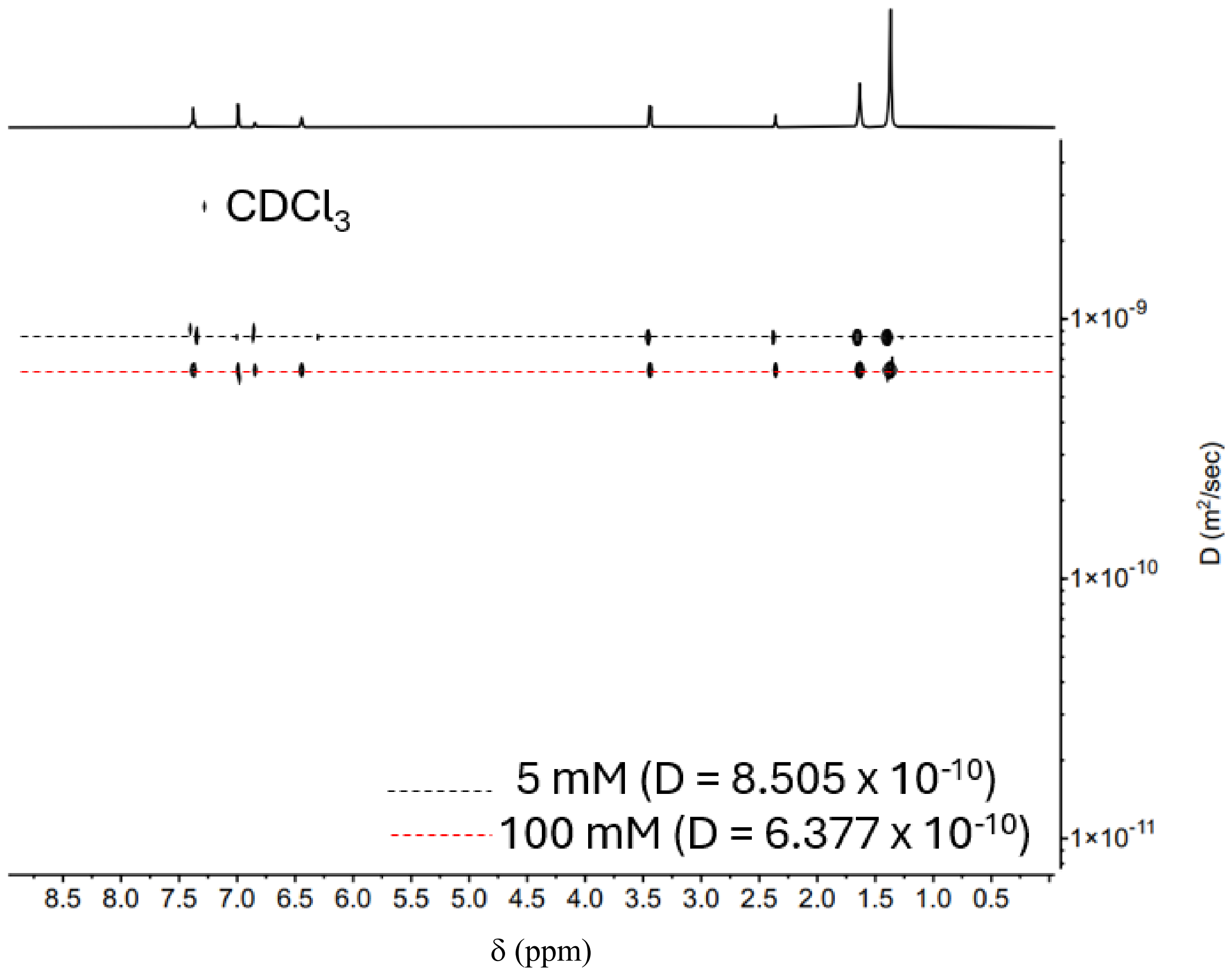

Supplementary Fig. 3 | Stacked Convection-compensated ^{1}H DOSY NMR Spectra (800 MHz, $CDCl_3$, 298 K) of SNAC at 5 mM and 100 mM Concentrations. A clear decrease in the apparent diffusion coefficient is observed with increasing SNAC concentration, consistent with the formation of dimers and larger oligomers observed by DLS (Supplementary Fig. 4) and all-atom C*p*HMD simulations (Supplementary Fig. 26).

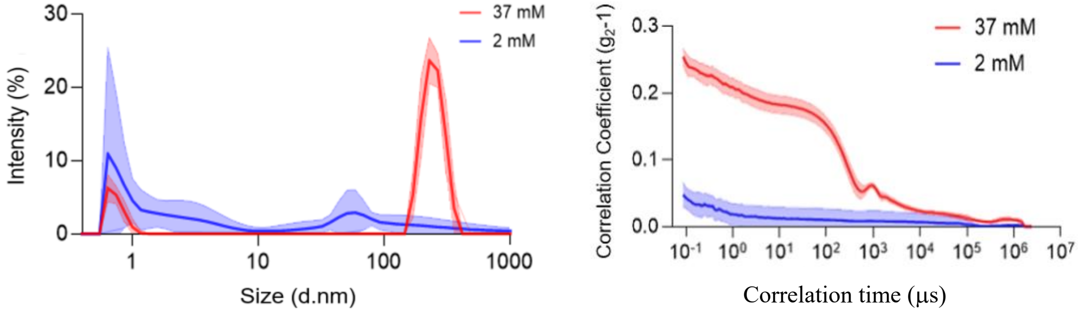

Supplementary Fig. 4 | DLS Spectra and Correlogram of SNAC in $CDCl_3$. DLS spectra and corresponding correlograms (298 K, mean ± standard deviation, $n = 8$ for 37 mM [SNAC] and $n = 9$ for 2 mM [SNAC] due to the exclusion of 3 outliers, as detailed in the data reporting summary, calculated using GraphPad Prism Version 10.2.3) of SNAC in $CDCl_3$ at 37 mM (red) and 2 mM (blue). The data demonstrates the formation of larger SNAC aggregates in this non-polar environment at the higher concentration. Source data are provided as a Source Data file.

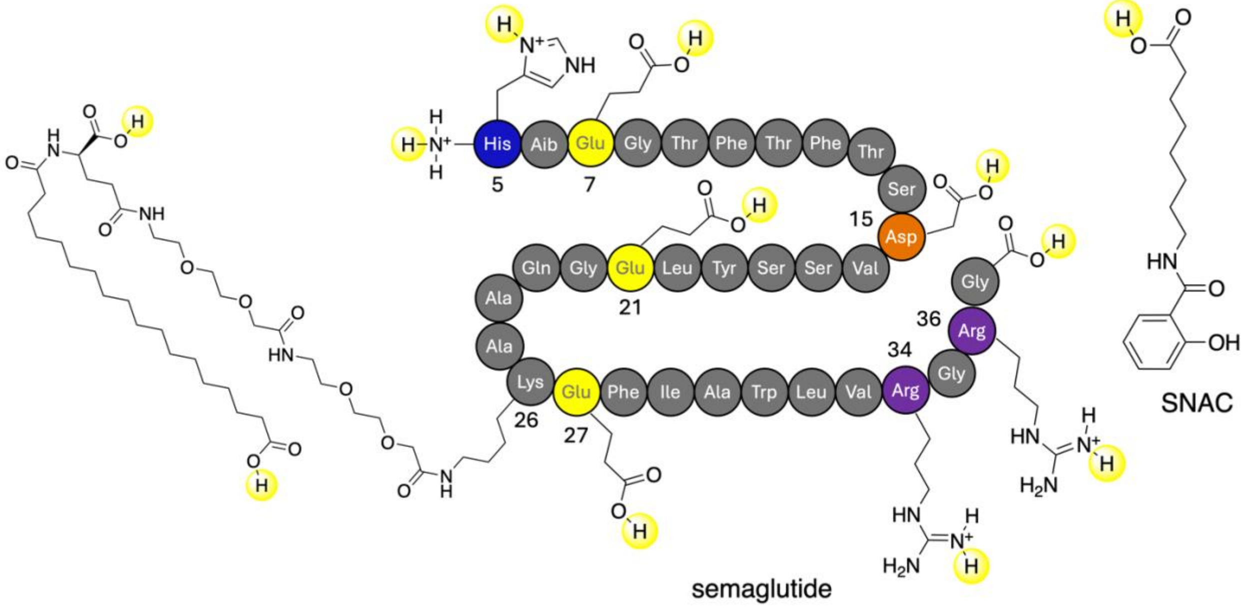

Supplementary Fig. 5 | Structures of Semaglutide and SNAC. All the ionizable protons in semaglutide and SNAC treated with λ-dynamics are highlighted in yellow.

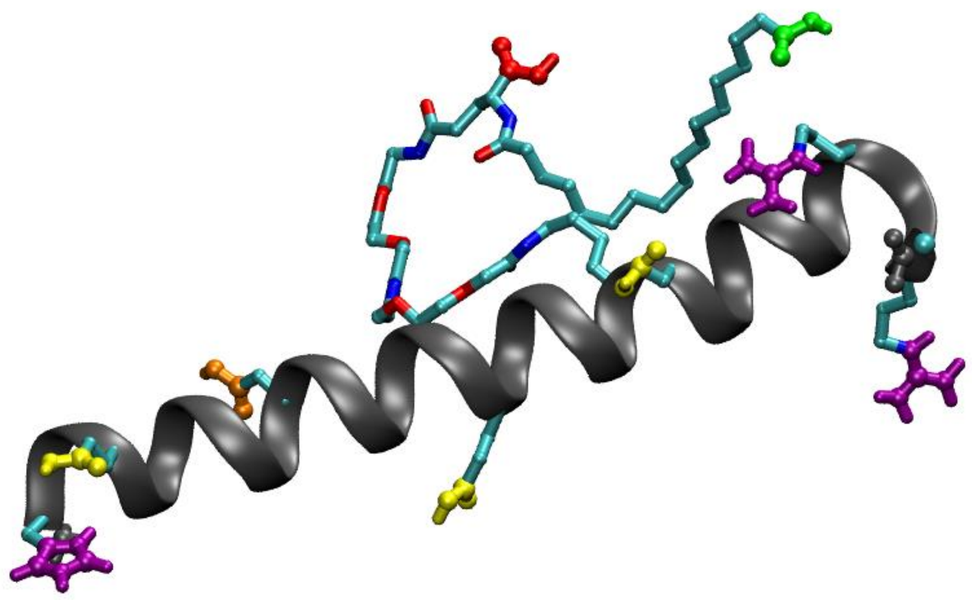

Supplementary Fig. 6 | 3D Structural Model of Semaglutide. The peptide backbone is shown with a ribbon representation. Hydrogen atoms are omitted for clarity. The ionizable side chains that were treated with λ-dynamics are highlighted in stick mode with their titratable atoms color-coded corresponding to Supplementary Fig. 5.

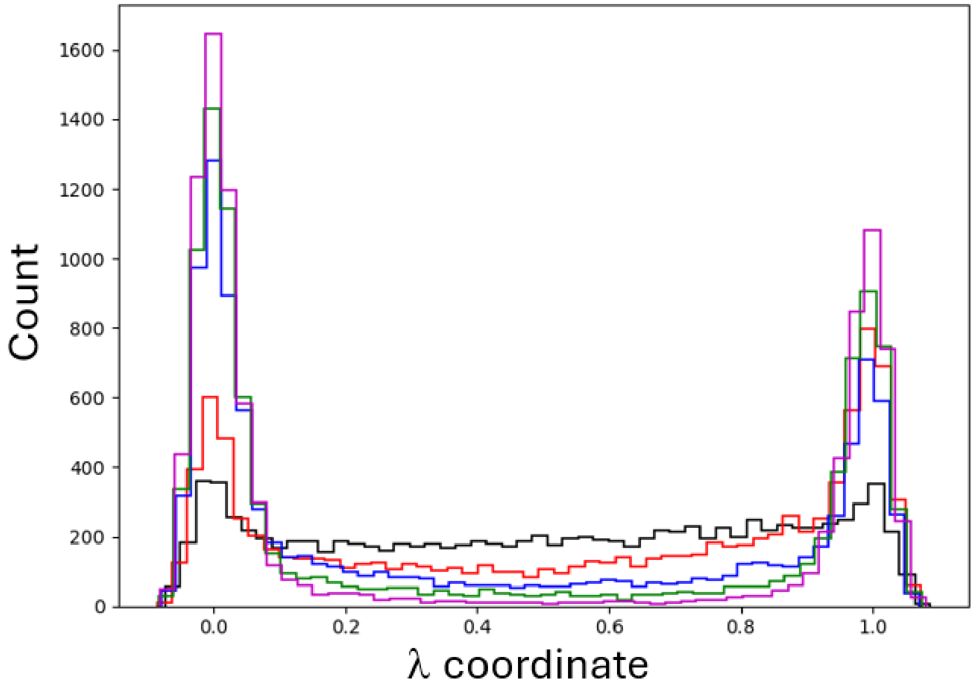

Supplementary Fig. 7 | Barrier Height Optimization of the C*p*HMD Biasing Potential for the Titratable Site in SNAC. The observed distribution of λ coordinates is influenced by the varying barrier heights of the biasing potential: 2.5 kJ/mol (black), 5.0 kJ/mol (gred), 7.5 kJ/mol (blue), 10.0 kJ/mol (green), and 12.5 kJ/mol (purple). Source data are provided as a Source Data file.

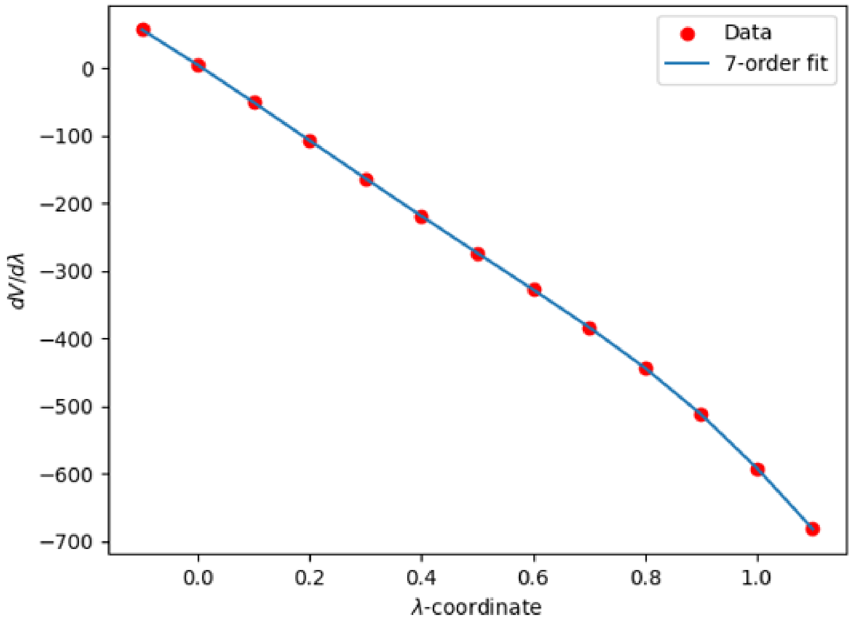

Supplementary Fig. 8 | Fit for the ∂V/∂λ C*p*HMD Coefficients for SNAC. Source data are provided as a Source Data file.

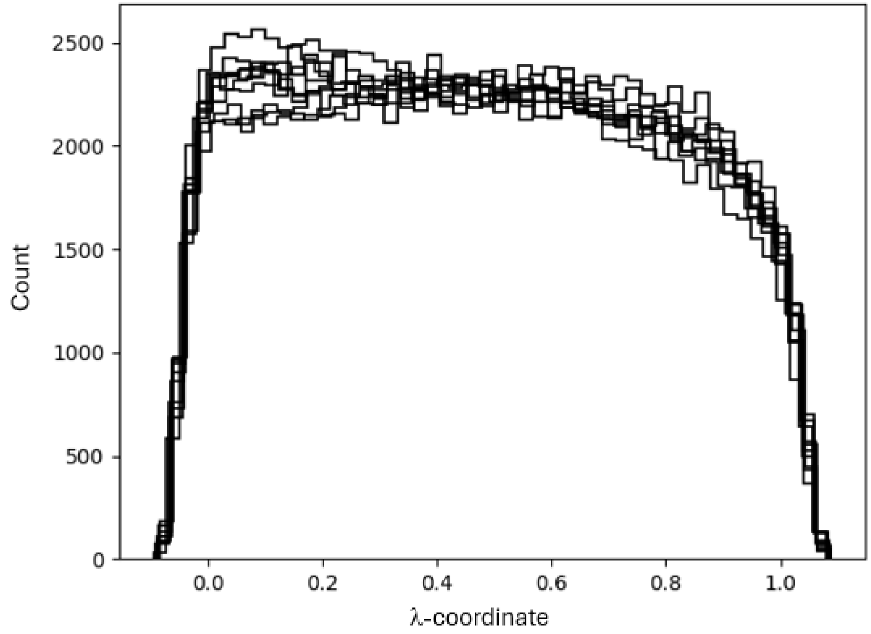

Supplementary Fig. 9 | Distribution of λ-Values Plotted for 10 100 ns Replica Validation Simulations of SNAC. Source data are provided as a Source Data file.

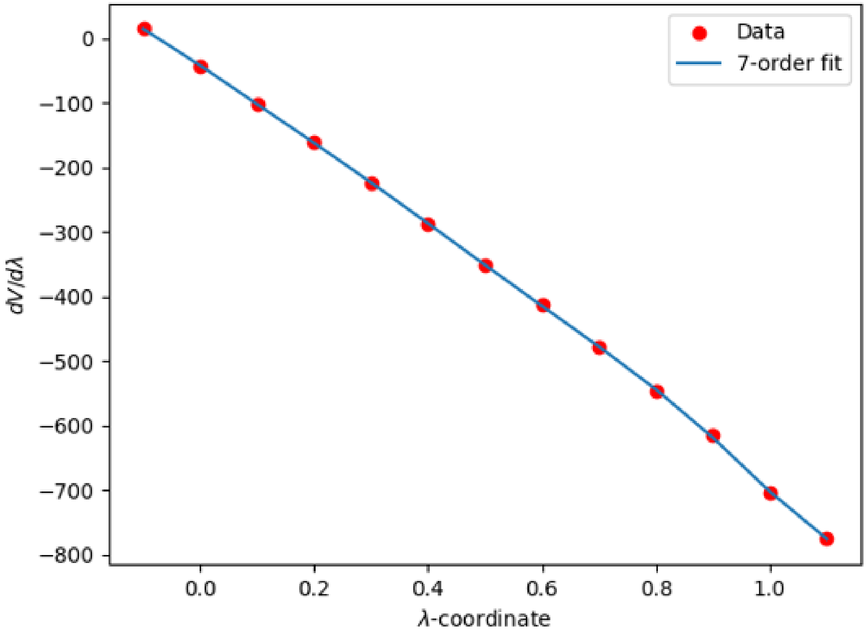

Supplementary Fig. 10 | Fit for the ∂V/∂λ C*p*HMD Coefficients for the SCH1 Residue. Source data are provided as a Source Data file.

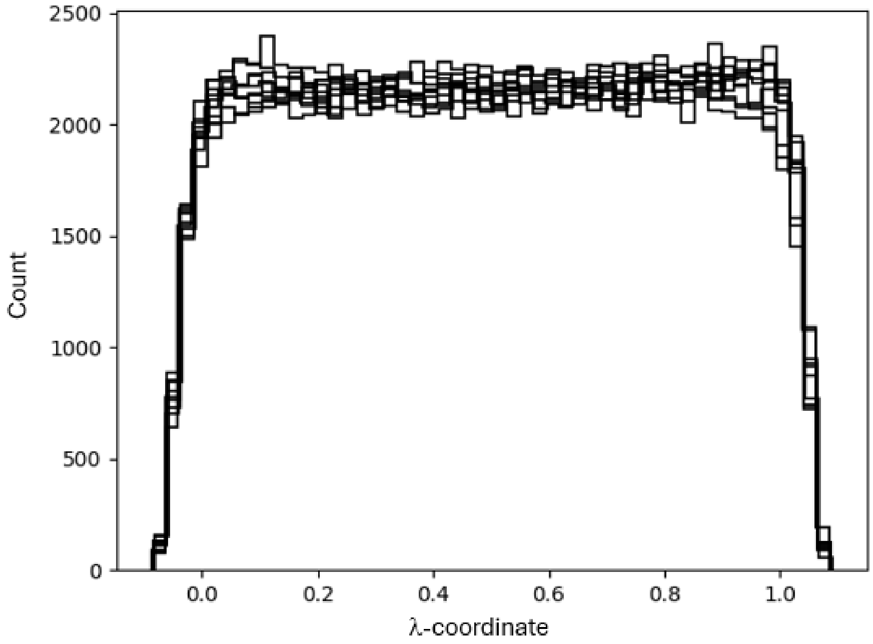

Supplementary Fig. 11 | Distribution of λ-Values Plotted for 10 100 ns Replica Validation Simulations of the SCH1 Residue. Source data are provided as a Source Data file.

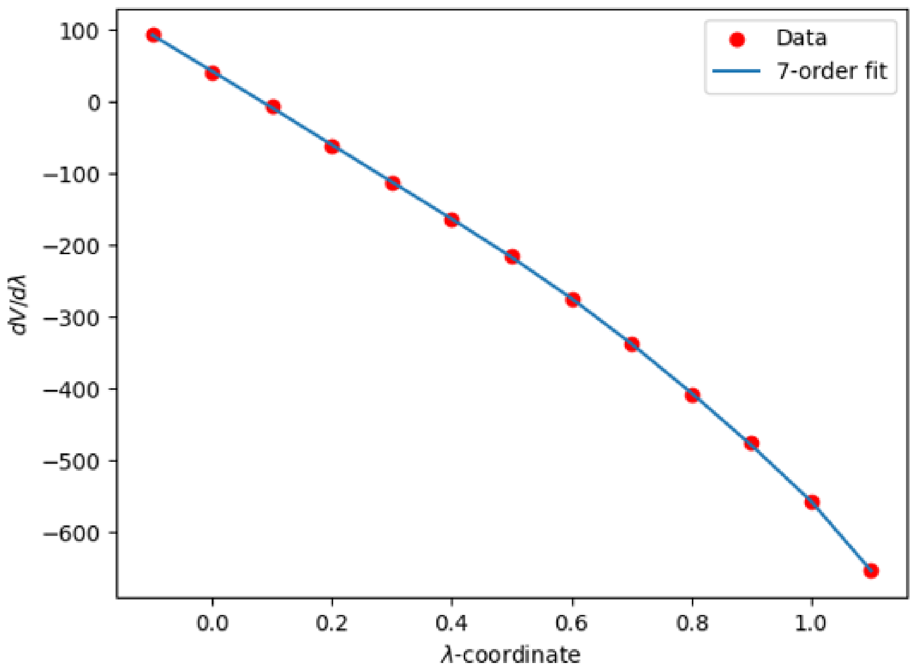

Supplementary Fig. 12 | Fit for the ∂V/∂λ C*p*HMD Coefficients for the SCH2 Residue. Source data are provided as a Source Data file.

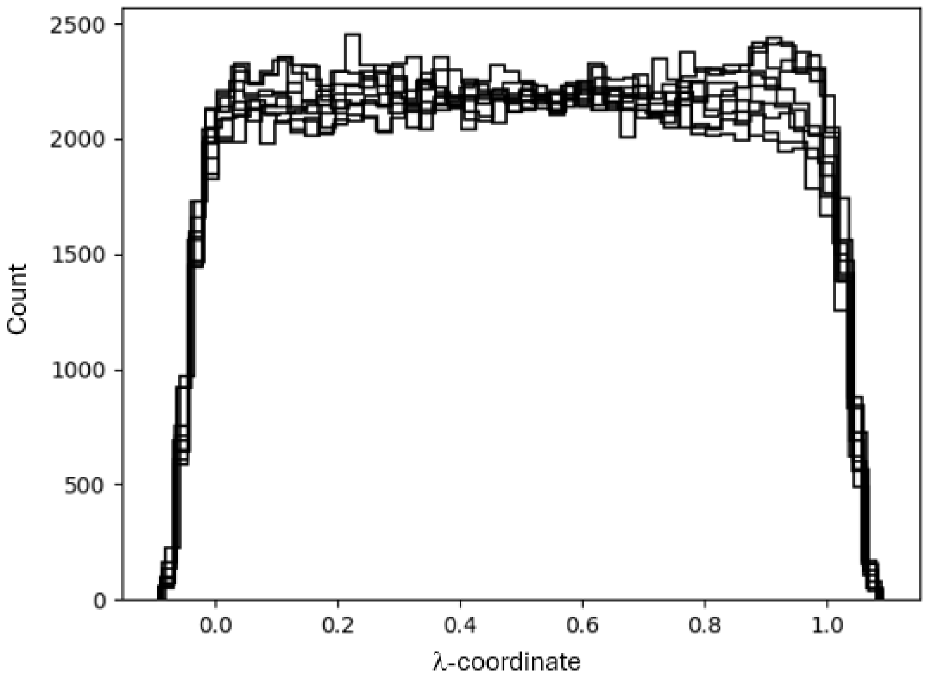

Supplementary Fig. 13 | Distribution of λ-Values Plotted for 10 100 ns Replica Validation Simulations of the SCH2 Residue. Source data are provided as a Source Data file.

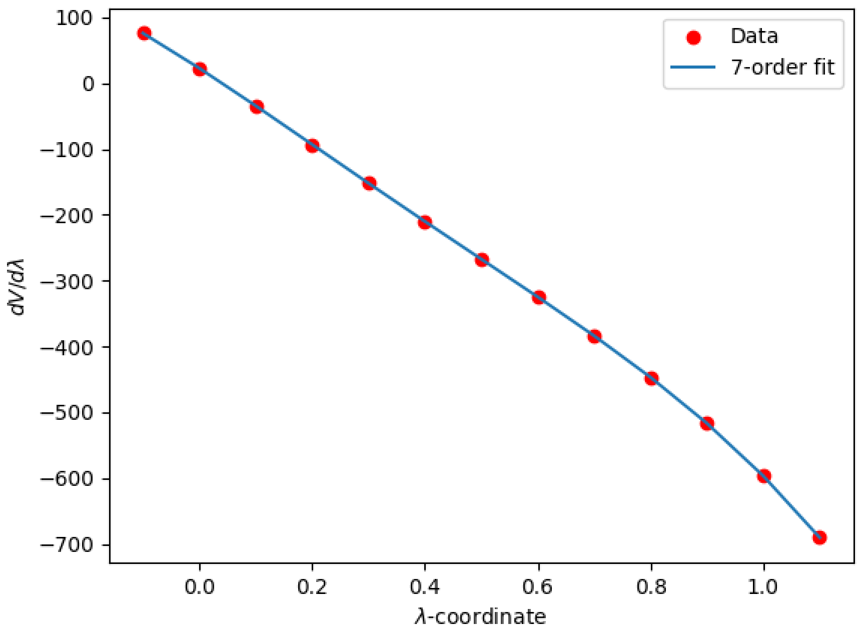

Supplementary Fig. 14 | Fit for the ∂V/∂λ C*p*HMD Coefficients for the OLET Residue. Source data are provided as a Source Data file.

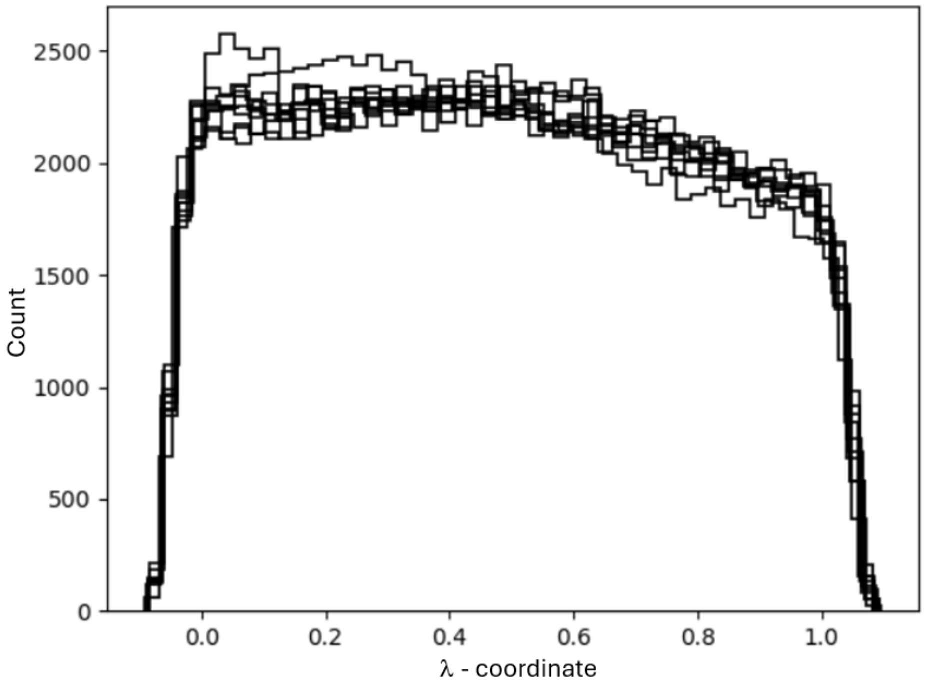

Supplementary Fig. 15 | Distribution of λ-Values Plotted for 10 100 ns Replica Validation Simulations of the OLET Residue. Source data are provided as a Source Data file.

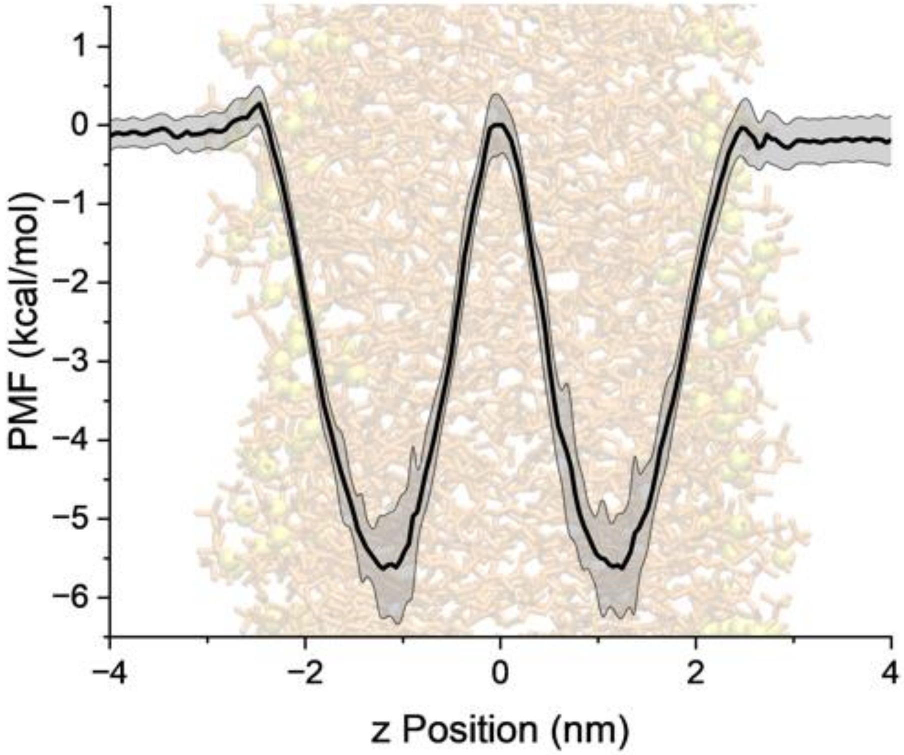

Supplementary Fig. 16 | Potential of Mean Force (PMF) Profile for pulling a SNAC molecule through a POPC Model Membrane. The PMF was obtained at 310.15K with SNAC, and the membrane (containing 64 POPC lipids per leaflet) was modeled with the CHARMM36 Force Field. The water layer contained 0.15 M NaCl. The free energy curve was obtained by umbrella sampling (with harmonic restraints on the z-coordinate — defined as the z-distance from the membrane center (COM of the phospholipid headgroups) to the COM of SNAC — with a force constant of 1000 kJ mol^{-1} nm^{-2}) with the center of mass of SNAC constrained to the umbrella sampling windows shown in Supplementary Fig. 17, followed by standard WHAM analysis. Shaded bands represent standard error estimates obtained with boostrapping analysis (n = 200) implemented in the GROMACS WHAM analysis software.[12] Source data are provided as a Source Data file.

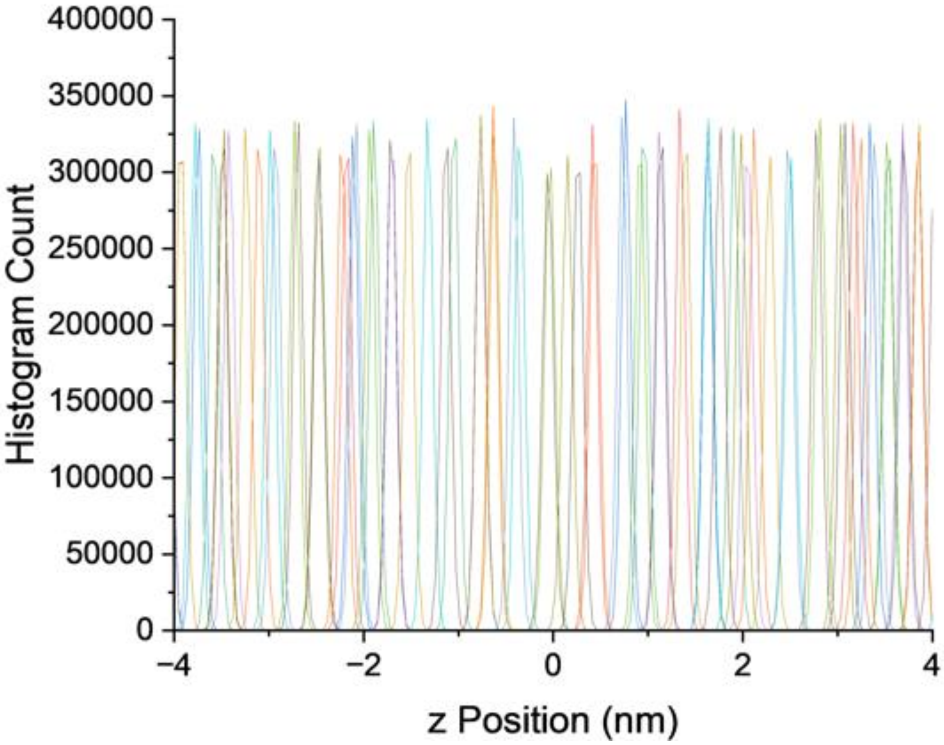

Supplementary Fig. 17 | Umbrella Sampling Windows used to Calculate the PMF Profile Shown in Supplementary Fig. 16. This data was obtained with standard molecular dynamics with window sampling lengths of 200 ns with harmonic restraints and a force constant of 1000 kJ mol^{-1} nm^{-2}. Initial conformations were obtained from the pulling simulations shown in Supplementary Video 1. Source data are provided as a Source Data file.

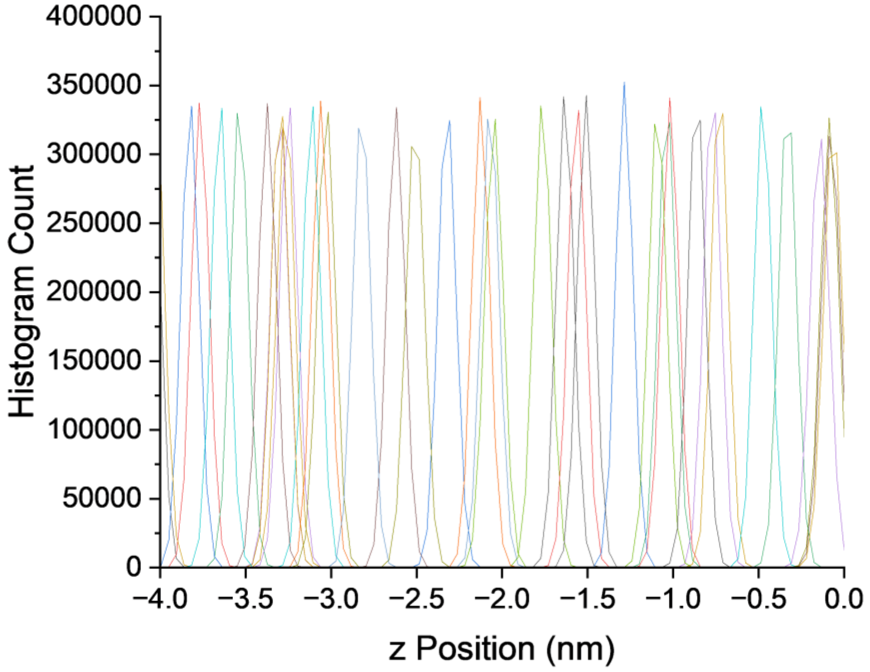

Supplementary Fig. 18 | Umbrella Sampling Windows used to Calculate the PMF Profile Shown in Fig. 2B. This data was obtained with the C*p*HMD model of SNAC as detailed in the Supplementary Methods section. Each umbrella sampling window was simulated for 200 ns with harmonic restraints and a force constant of 1000 kJ mol^{-1} nm^{-2}. Source data are provided as a Source Data file.

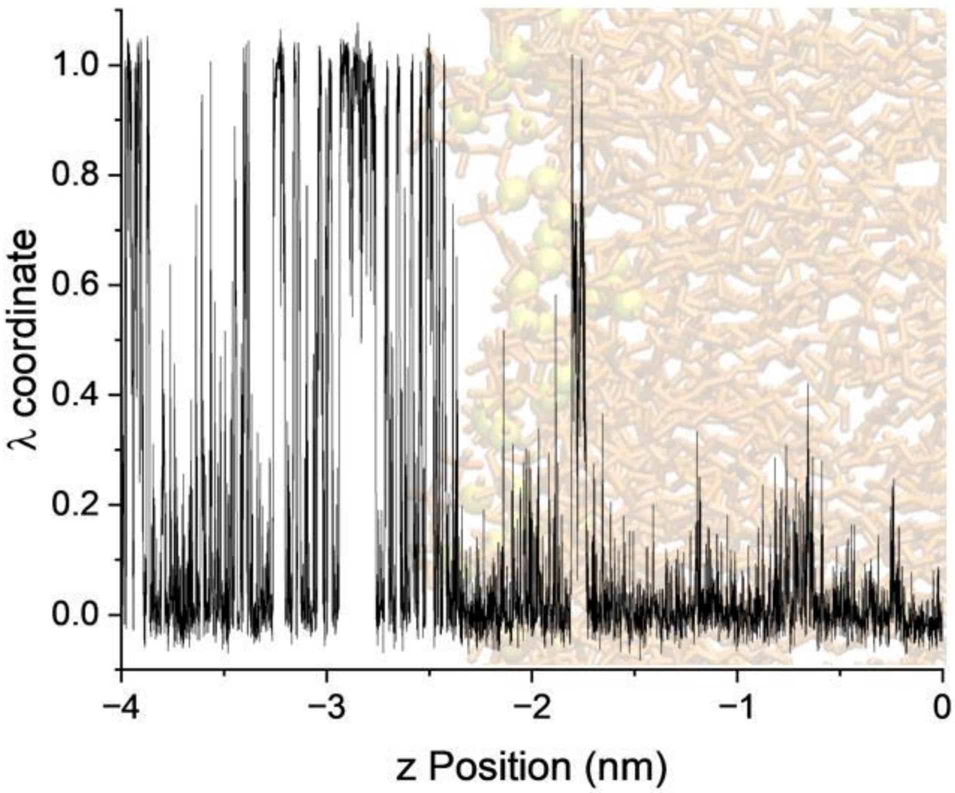

Supplementary Fig. 19 | λ-Coordinate Trajectory for One SNAC Molecule Being Pulled from the Center of a POPC Model Membrane into the Water Layer. The membrane model consisted of 64 POPC lipids in each leaflet, while the water layer contained 0.15 M NaCl at a *p*H of 5. In this simulation, we found that SNAC remained protonated in the nonpolar environment of the membrane interior but then demonstrated dynamic protonation/deprotonation behavior once it was pulled into the water layer. Source data are provided as a Source Data file.

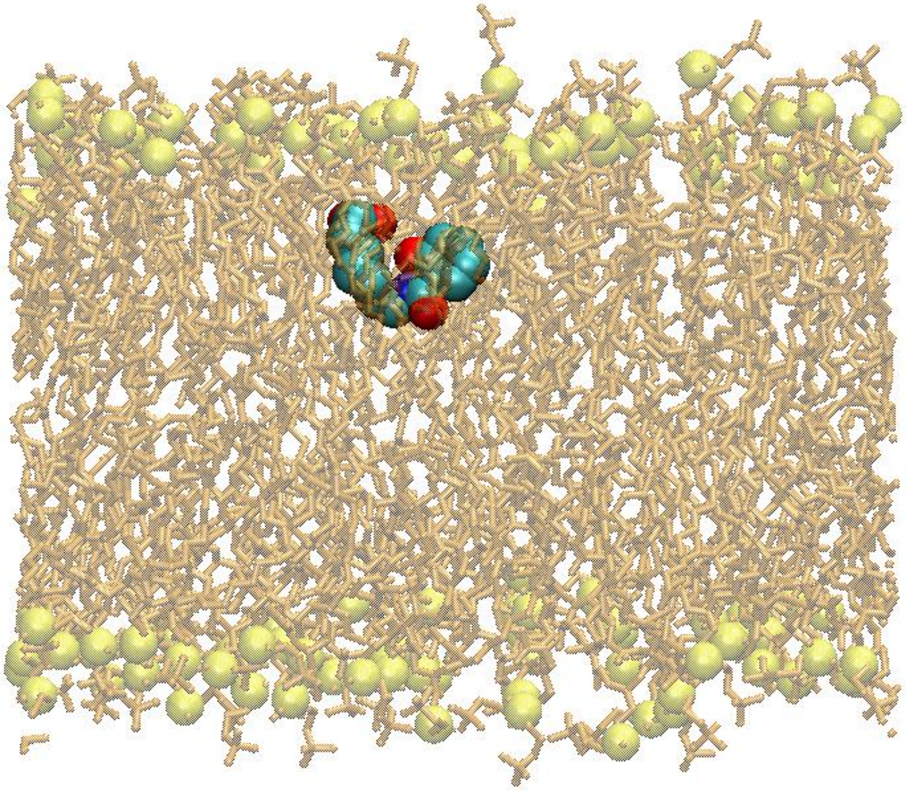

Supplementary Fig. 20 | Snapshot of the SNAC in a POPC Membrane Model Described in Fig. 2B (*z*-Position = –1.1 nm). The corresponding trajectory for this process is shown in Supplementary Video 1.

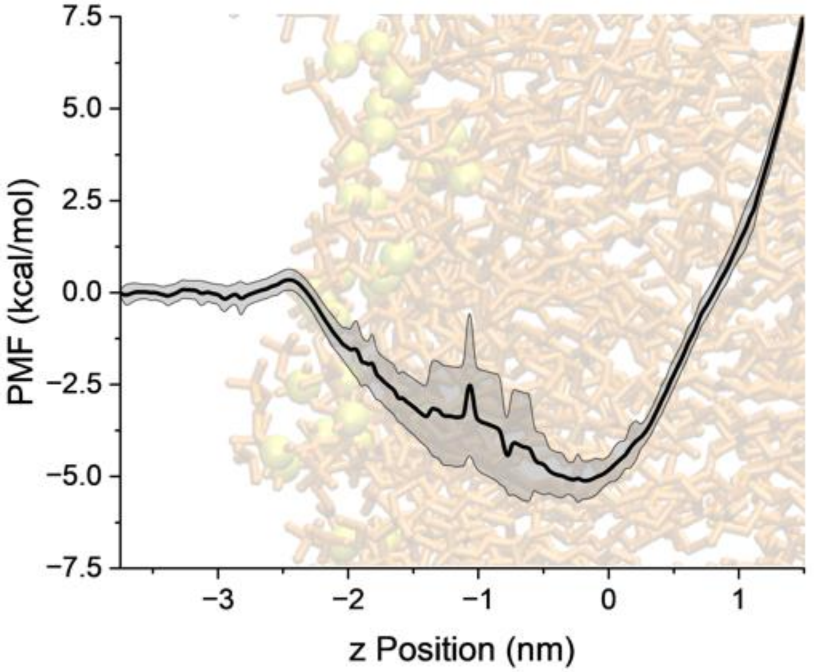

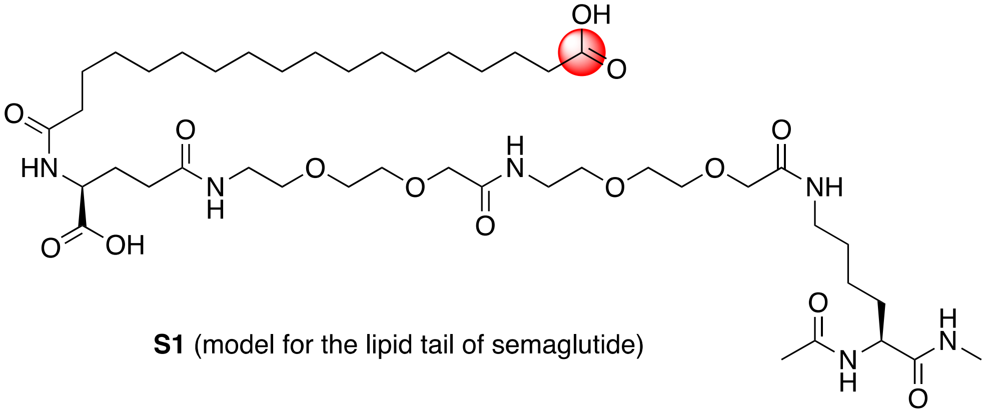

S1 (model for the lipid tail of semaglutide)

Supplementary Fig. 21 | Potential of Mean Force (PMF) Profile for Insertion of the Semaglutide Lipid Tail into a POPC Model Membrane. The PMF was obtained at 310.15K with the semaglutide lipid tail modeled as compound **S1** with the CHARMM36 Force Field. The water layer contained 0.15 M NaCl and the membrane was built with 64 POPC lipids per leaflet. The free energy curve was obtained by umbrella sampling (with the z-distance from the POPC lipid head groups of the carboxylic acid end tail (highlighted in red) of the semaglutide tail constrained to the umbrella sampling windows shown in Supplementary Fig. 24, followed by standard WHAM analysis. The methyl caps of the model tail of semaglutide were restrained with a 1000 kJ mol^{-1} nm^{-1} harmonic position restraint during the initial pulling simulation, which was removed during umbrella sampling. Harmonic restraints with a force constant of 1000 kJ mol^{-1} nm^{-2} were used for all umbrella sampling windows. Shaded bands represent standard error estimates obtained with boostrapping analysis (n = 200) implemented in the GROMACS WHAM analysis software.[12] Source data are provided as a Source Data file.

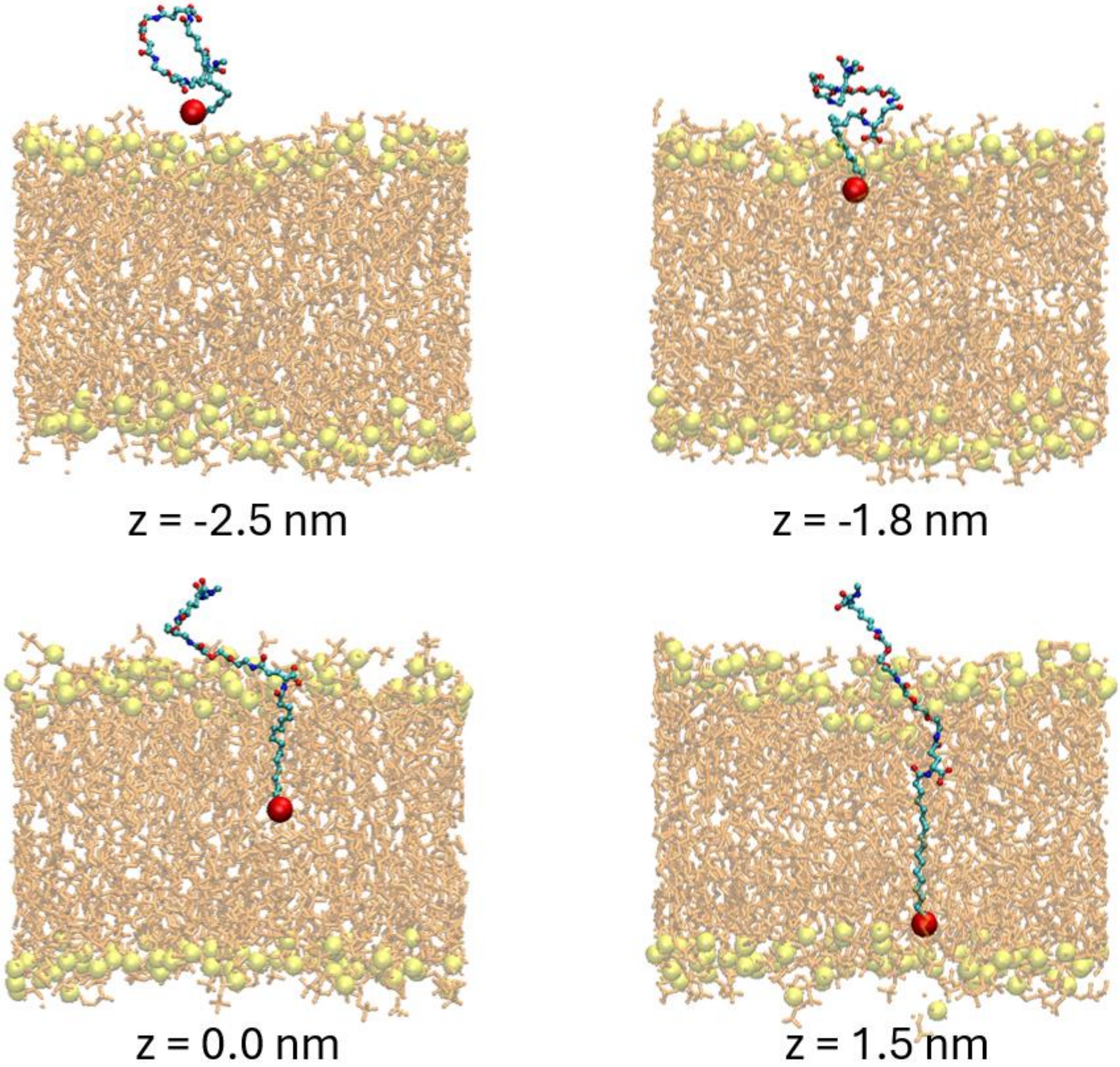

Supplementary Fig. 22 | Representative Snapshots for the PMF Profile Shown in Supplementary Fig. 21. The z-distance from the membrane center (COM of the phospholipid headgroups) of the COOH anchor (the atom highlighted in red in Supplementary Fig. 21 is labeled for each snapshot. For a movie of the corresponding trajectory, see Supplementary Video 6.

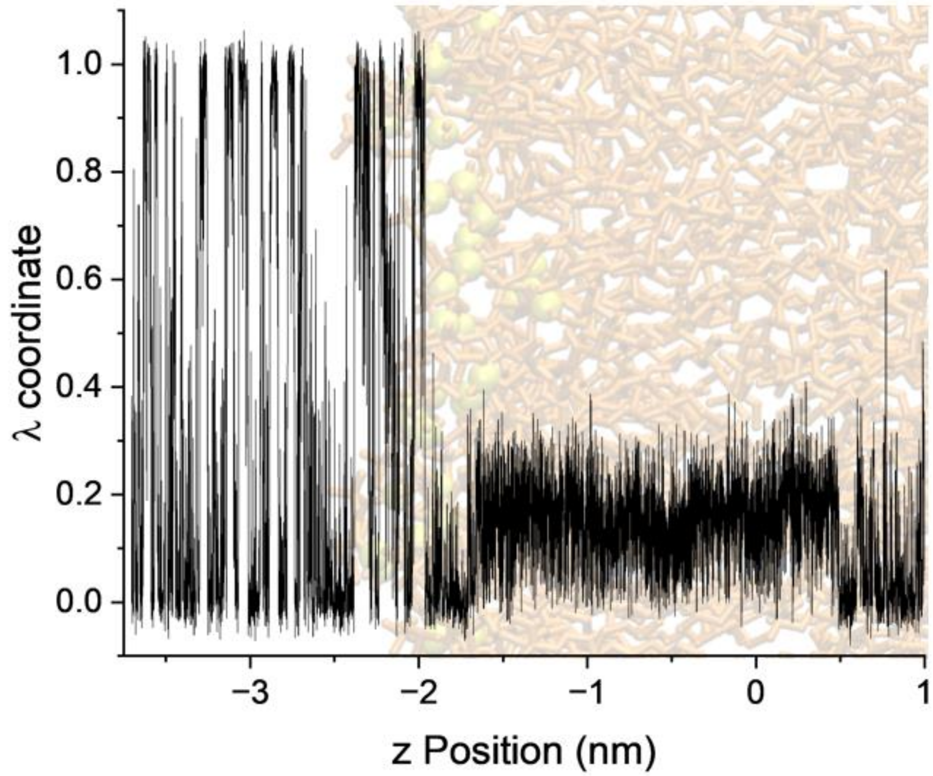

Supplementary Fig. 23 | λ-Coordinate Trajectory for Pulling Semaglutide's Lipid Tail into the Membrane. See Supplementary Fig. 22 for corresponding simulation snapshots. While insertion of the lipid tail into the membrane is favorable overall as shown by the PMF in Supplementary Fig. 21, pulling semaglutide's lipid tail completely to the other side of the membrane positioned the ionizable site of the γGlu-2xOEG linker inside the lipid bilayer, which ultimately lead to an increase of the free energy by ~8 kcal mol^{-1} toward the end of the PMF profile shown in Supplementary Fig. 21. Source data are provided as a Source Data file.

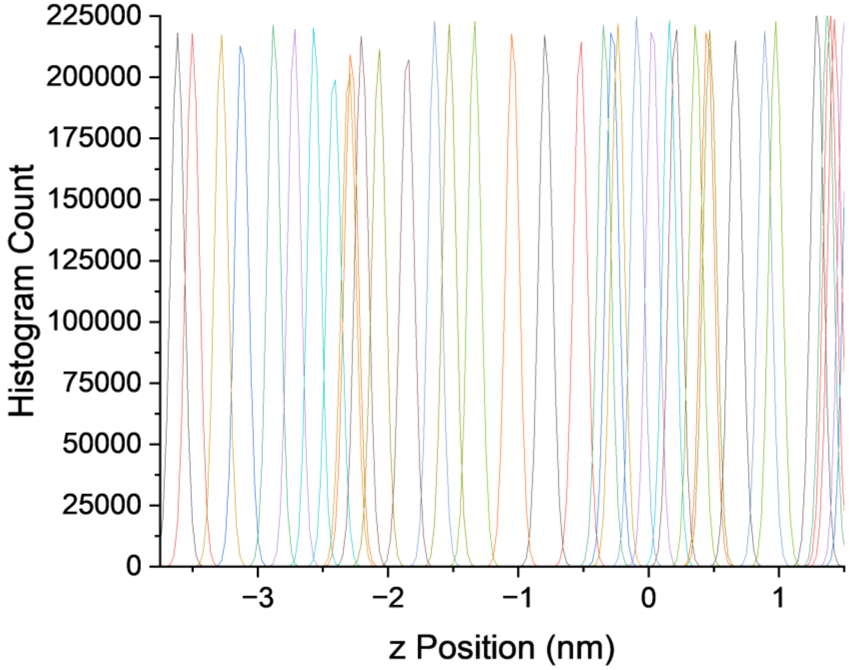

Supplementary Fig. 24 | Umbrella Sampling Windows used to Calculate the PMF Profile Shown in Supplementary Fig. 21. This data was obtained with the C*p*HMD model as detailed in the Supplementary Methods section. Sampling windows were collected for 200 ns with harmonic restraints and a force constant of 1000 kJ mol^{-1} nm^{-2}. Source data are provided as a Source Data file.

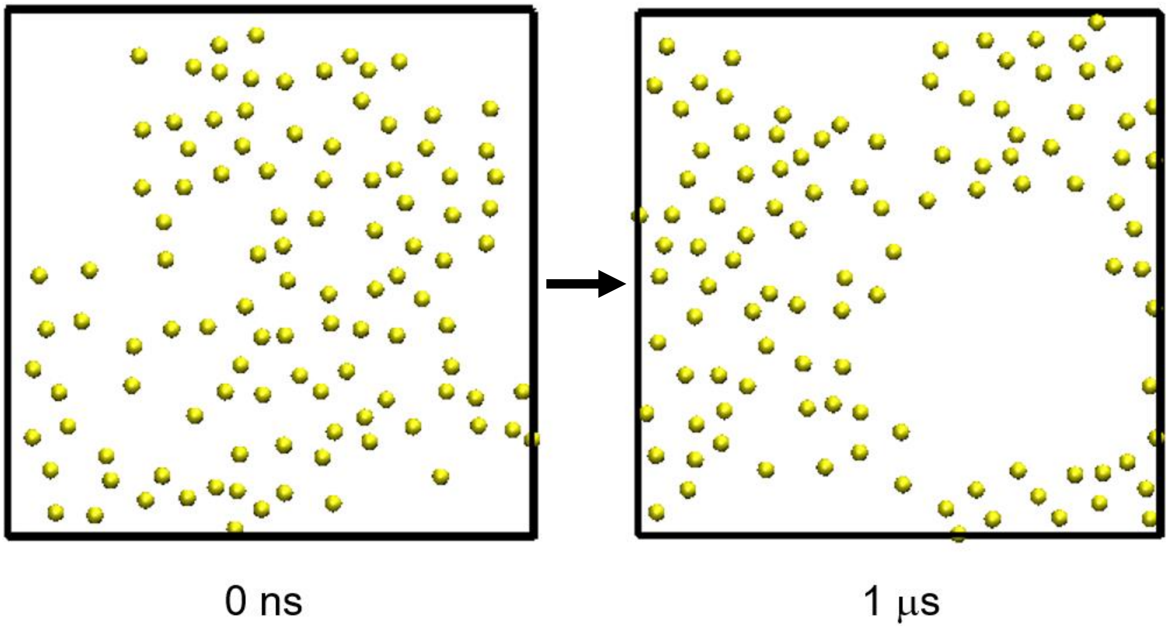

Supplementary Fig. 25 | Top-View of the Membrane-Defects Formed by SNAC-Incorporation into the Membrane. Representative snapshots are shown highlighting how SNAC influences the position of the phospholipid head groups (yellow) over the span of the 1-μs simulation of SNAC in the membrane. See Fig. 6 for a side-view of these membrane defects. As more SNAC aggregates in the membrane, the C*p*HMD simulations demonstrate that the SNAC-filled defects are expanding, which ultimately creates openings in the phospholipid head group barrier of the lipid bilayer. Semaglutide can then start to diffuse through these SNAC-filled openings, with a "quicksand-like" mechanism (as discussed in the main text). For a corresponding video of this process, see Supplementary Video 15.

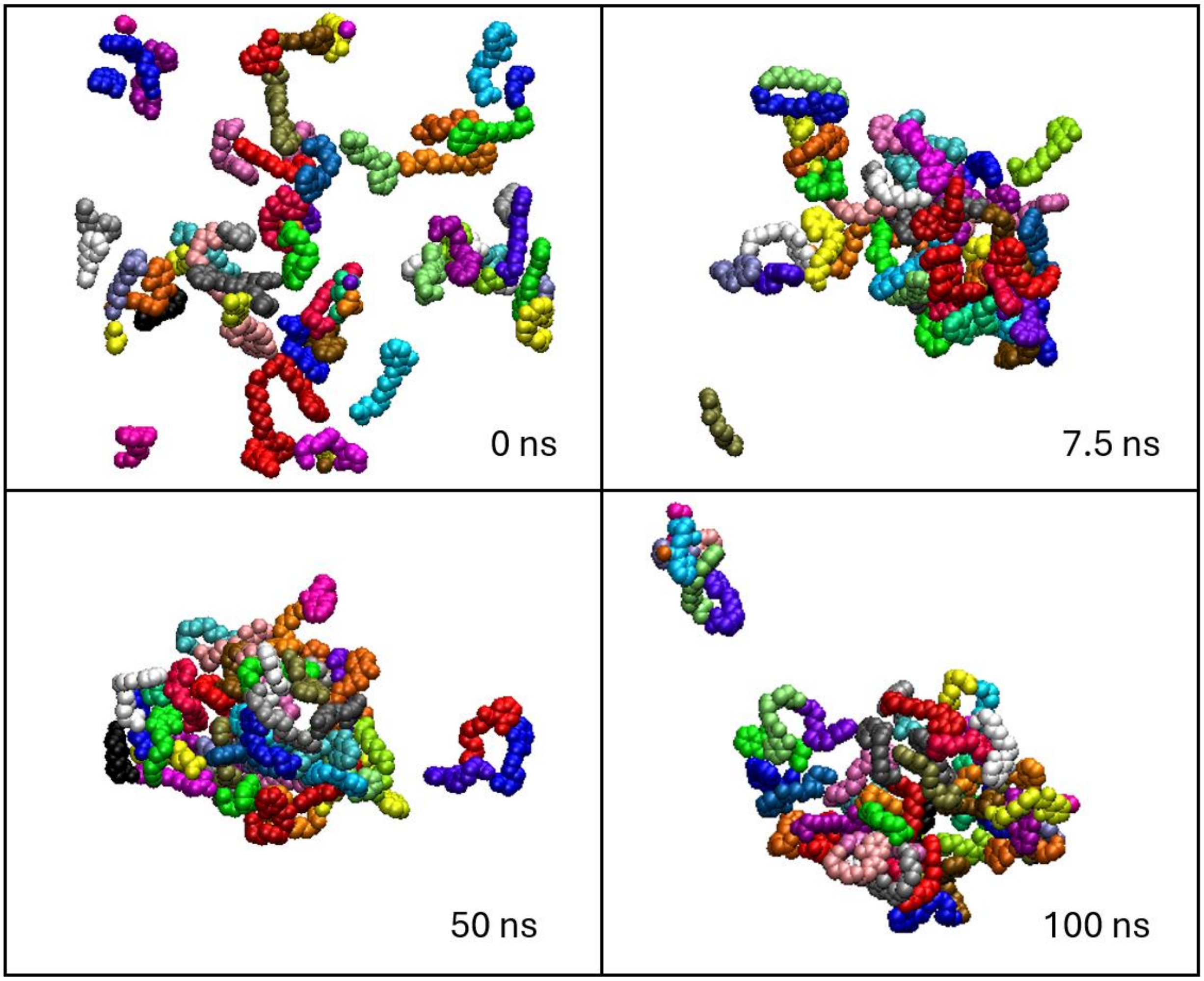

Supplementary Fig. 26 | Dynamic Aggregation of SNAC in CH_2Cl_2 as a Model for the Interior of a Lipid Bilayer Membrane. The figure shows representative snapshots of a 100-ns C*p*HMD simulation of 50 SNAC molecules, conducted at 310.15 K in a CH_2Cl_2 solvent box. SNAC molecules are color-coded to highlight the dynamic nature of the aggregate that forms in this non-polar environment. Hydrogens are omitted for clarity. Transient smaller aggregates of 2–4 SNAC molecules dynamically dissociate and rejoin with main cluster over the course of the simulation as shown Supplementary Video 3.

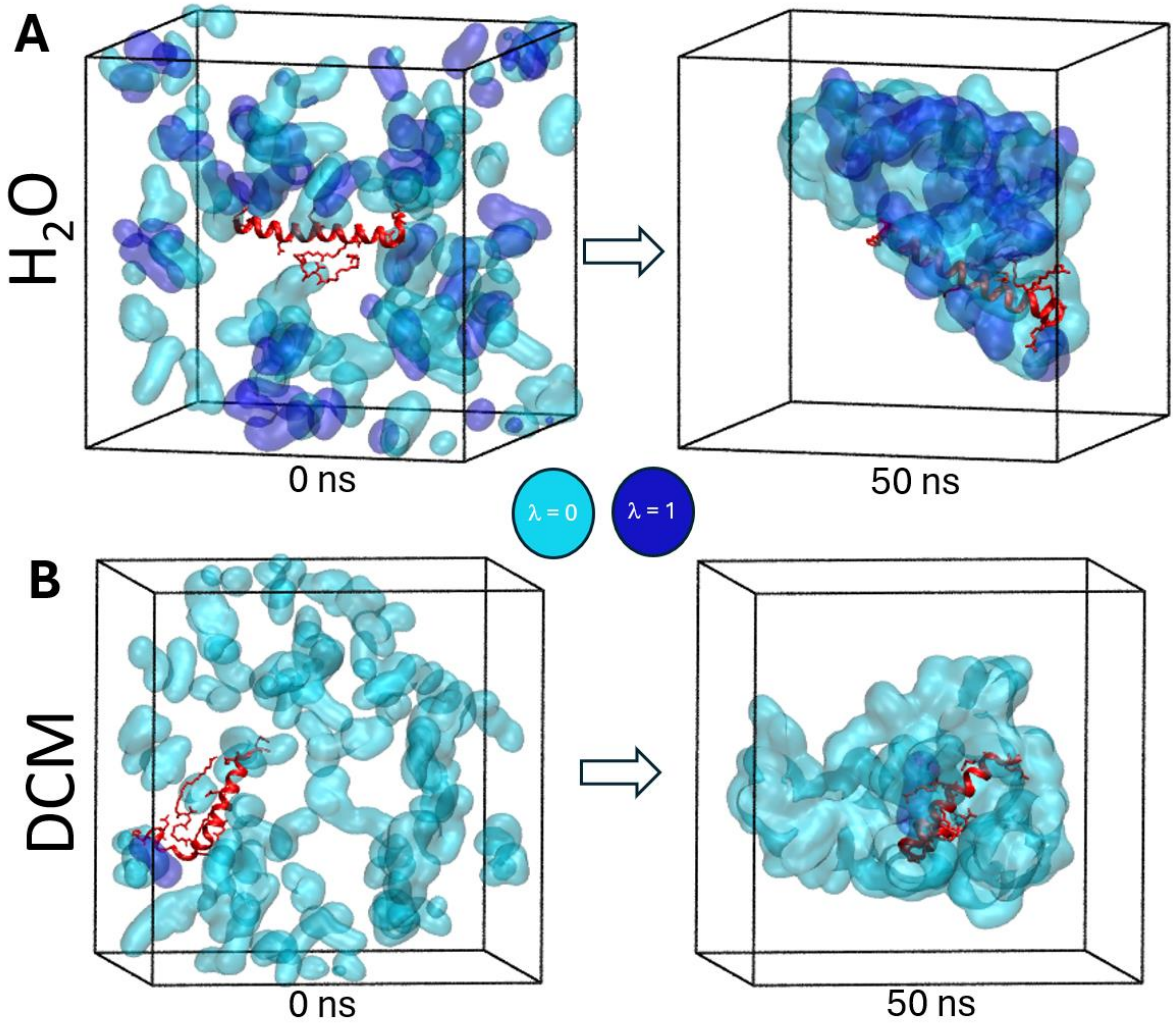

Supplementary Fig. 27 | Comparison of SNAC Aggregation with Semaglutide in Different Environments. The figure displays representative snapshots of unbiased C*p*HMD simulations with SNAC and semaglutide (50:1) in **(A)** aqueous solution (0.15 M NaCl, 310.15 K) and **(B)** a nonpolar solvent (CH_2Cl_2) as a simple, experimentally testable model for the interior, hydrophobic part of a membrane. The protonation states for the SNAC molecules are color-coded based on the following color scheme: Deprotonated SNAC ($\lambda > 0.5$) is shown in blue and protonated SNAC ($\lambda < 0.5$) in cyan. Overall, the data shows that in water, SNAC aggregates around the non-polar portions of semaglutide, which leaves the ionizable residues of semaglutide exposed to the solvent. However, in a non-polar environment, SNAC stabilizes semaglutide by aggregating around the peptide's polar residues, with the aggregates remaining more fluid than in water. This observed difference in aggregation behavior between the two environments, which also includes the hydrogen-bonded aggregates in the nonpolar environment being more fluid than the hydrophobic aggregates in water, facilitates membrane permeation as discussed in the main text. Representative videos of the corresponding trajectories are shown in Supplementary Videos 4 and 5.

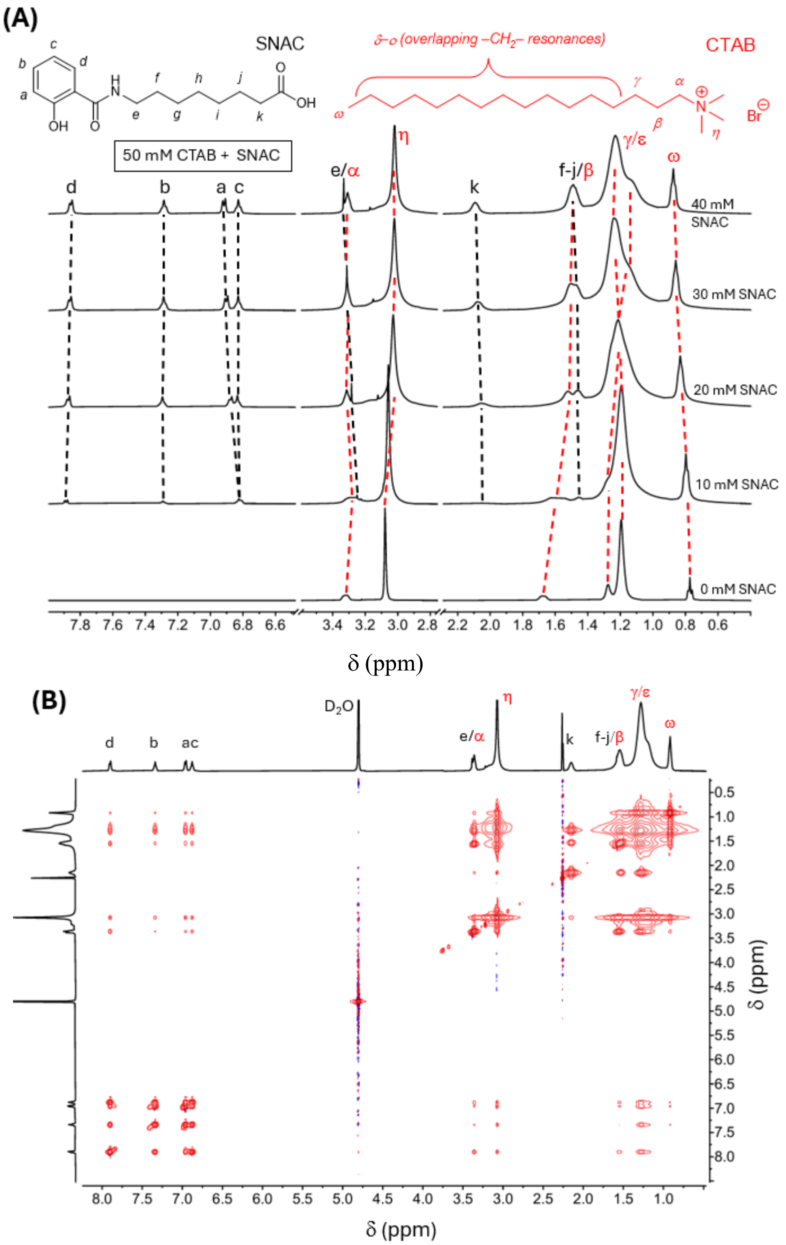

Supplementary Fig. 28 | Concentration-dependent NMR Data for CTAB Micelles and SNAC. (A) Stacked 1H NMR spectra (500 MHz, D2O, 298 K, referenced to the DSS internal standard) for CTAB (50 mM) and SNAC in different concentrations, with all NMR resonances assigned. The broadening and shifting of the SNAC resonances in the aromatic region, as well as of the CTAB resonances in the aliphatic region, support the computationally observed (see Fig. 5) aggregation of CTAB with SNAC, which is also reminiscent of what was previously observed for CTAB interacting with *p*-toluenesulfonate/*p*-toluenesulfonic acid.[13] **(B)** Full 1H-1H NOESY NMR spectrum (500 MHz, D2O, 298 K) of 40 mM SNAC and its conjugate acid (4:1 molar ratio) and 50 mM CTAB.

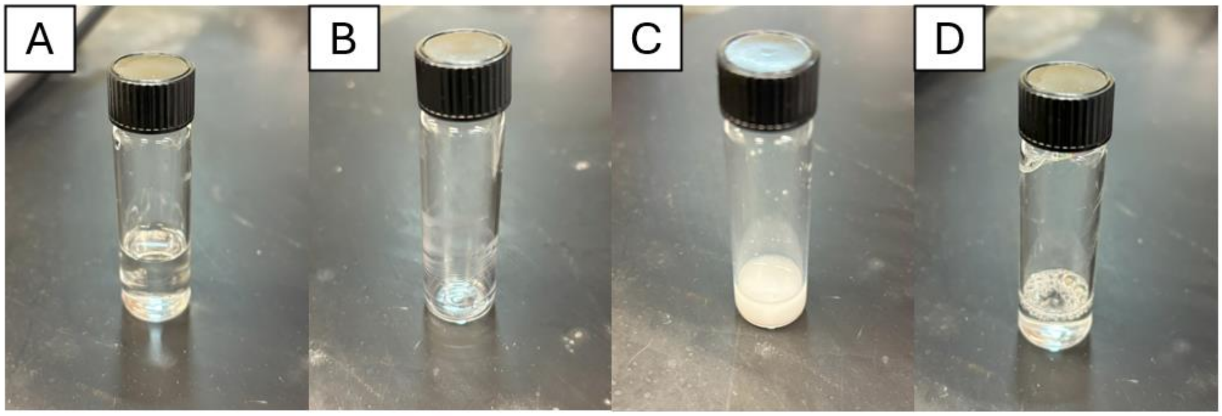

Supplementary Fig. 29 | Images to Highlight the Increased Solubility of the Conjugate Acid of SNAC in the Presence of CTAB Micelles. (A) SNAC and its conjugate acid (4:1 molar ratio, 40 mmol total SNAC) were dissolved in 2.0 mL of MeOH. **(B)** A thin layer of SNAC film is formed in the scintillation vial after removing MeOH under reduced pressure. **(C)** Adding 1.0 mL of DI water to the SNAC film results in a cloudy solution due to the low solubility of the conjugate acid of SNAC in water. **(D)** Adding 1.0 mL of a 50 mM solution of CTAB in DI water results in a clear solution. Therefore, the presence of CTAB micelles increases the solubility of the conjugate acid of SNAC in an aqueous environment, consistent with our computational models (Fig. 5 and Supplementary Fig. 30), which also show SNAC and CTAB micelles co-aggregating spontaneously in an aqueous environment.

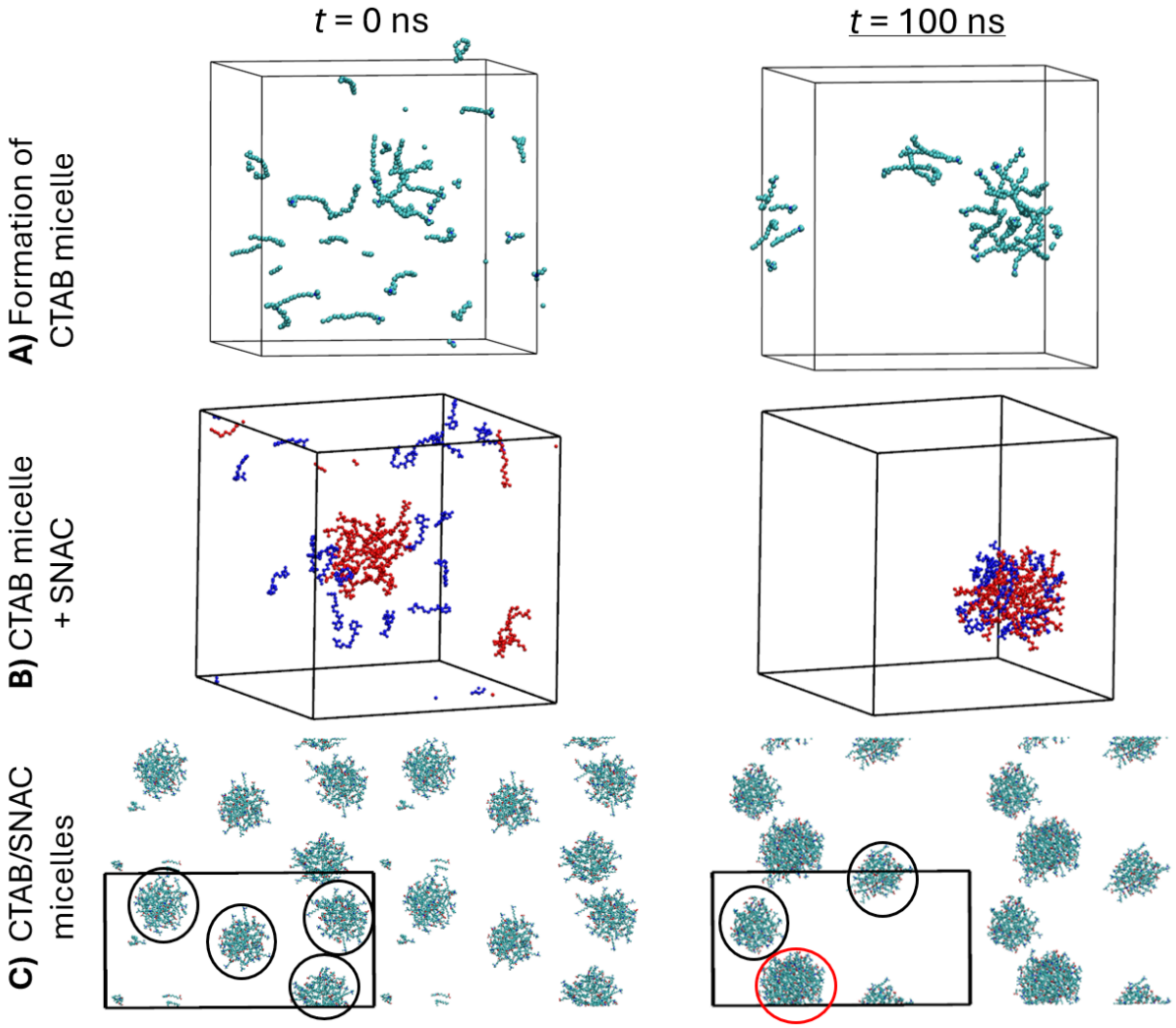

Supplementary Fig. 30 | Snapshots of MD simulations of CTAB and SNAC Demonstrate the Aggregation of SNAC in CTAB Micelles. (A) Standard MD simulation of 25 molecules of CTAB (100 mM) forming a micelle. **(B)** C*p*HMD (*p*H = 5.6) simulation of 20 molecules of SNAC (blue: 40 mM) randomly placed around the CTAB (red: 25 molecules, 50 mM) micelle demonstrates the incorporation of SNAC into the CTAB micelle. **(C)** C*p*HMD (*p*H = 5.6) of four CTAB/SNAC micelles (circled in black) shows two micelles coming together to form a larger micelle (circled in red) that demonstrates SNAC clustering in the micelle. For a zoomed-in image of the final, assembled micelle, see Fig. 5B. These processes are also visualized in Supplementary Videos 7–9.

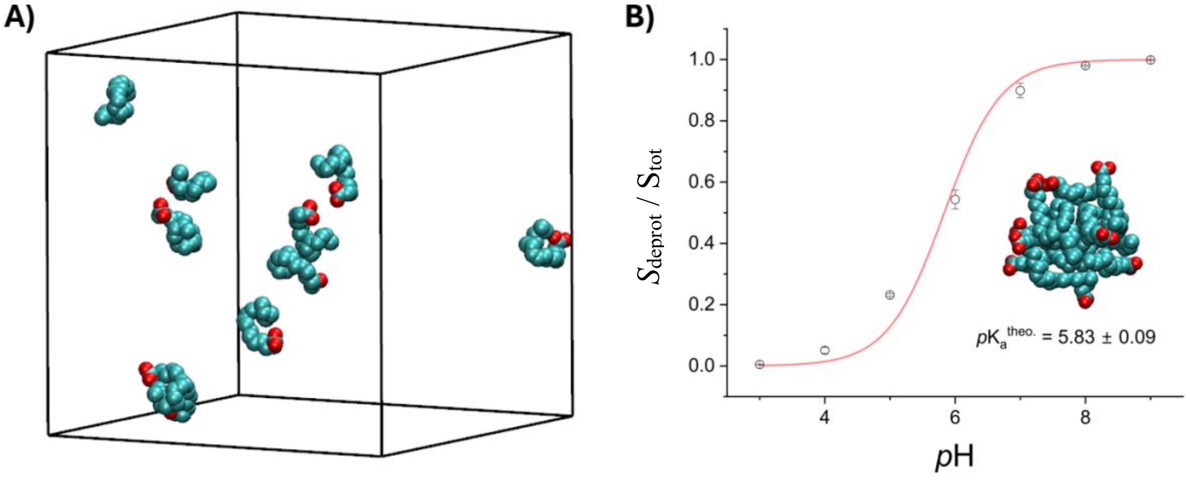

Supplementary Fig. 31 | Validation of the C*p*HMD Approach in a Membrane-like Environment by Predicting the Apparent *p*K_a Value of an Oleic Acid Micelle Model System. **(A)** Oleic acid (10 molecules, 40 mM) was randomly placed in a water box with 100 buffer particles and allowed to self-assemble for 100 ns (*n* = 3) in different *p*H environments ranging from *p*H 3 to 9 (solvent and buffer molecules are not shown in the figure for clarity). The ∂V/∂λ C*p*HMD coefficients for oleic acid were obtained from simulations of monomeric oleic acid in water (see also Supplementary Figs. 14 and 15) containing 0.15 M NaCl. All replicas of this simulation resulted in the formation of micelles. **(B)** Plot of the fraction of λ-values corresponding to deprotonated sites (S_{deprot} / S_{tot}) among all oleic acid residues in the simulation box (mean ± standard deviation, *n* = 3). The distribution of λ-coordinates was determined and averaged across all three replicas to produce a titration curve, which was then fit using the Henderson-Hasselbalch equation. The apparent *p*K_a of this simplified system (*p*K_a^{theo} = 5.8) matches reasonably well with the apparent *p*K_a of oleic acid/glycerol monooleate systems containing 22% w/w oleic acid (*p*K_a = 6.0). This serves as proof of concept for how this methodology can accurately model the influence of a membrane-like environment on the *p*K_a values of carboxylic acids. Source data are provided as a Source Data file.

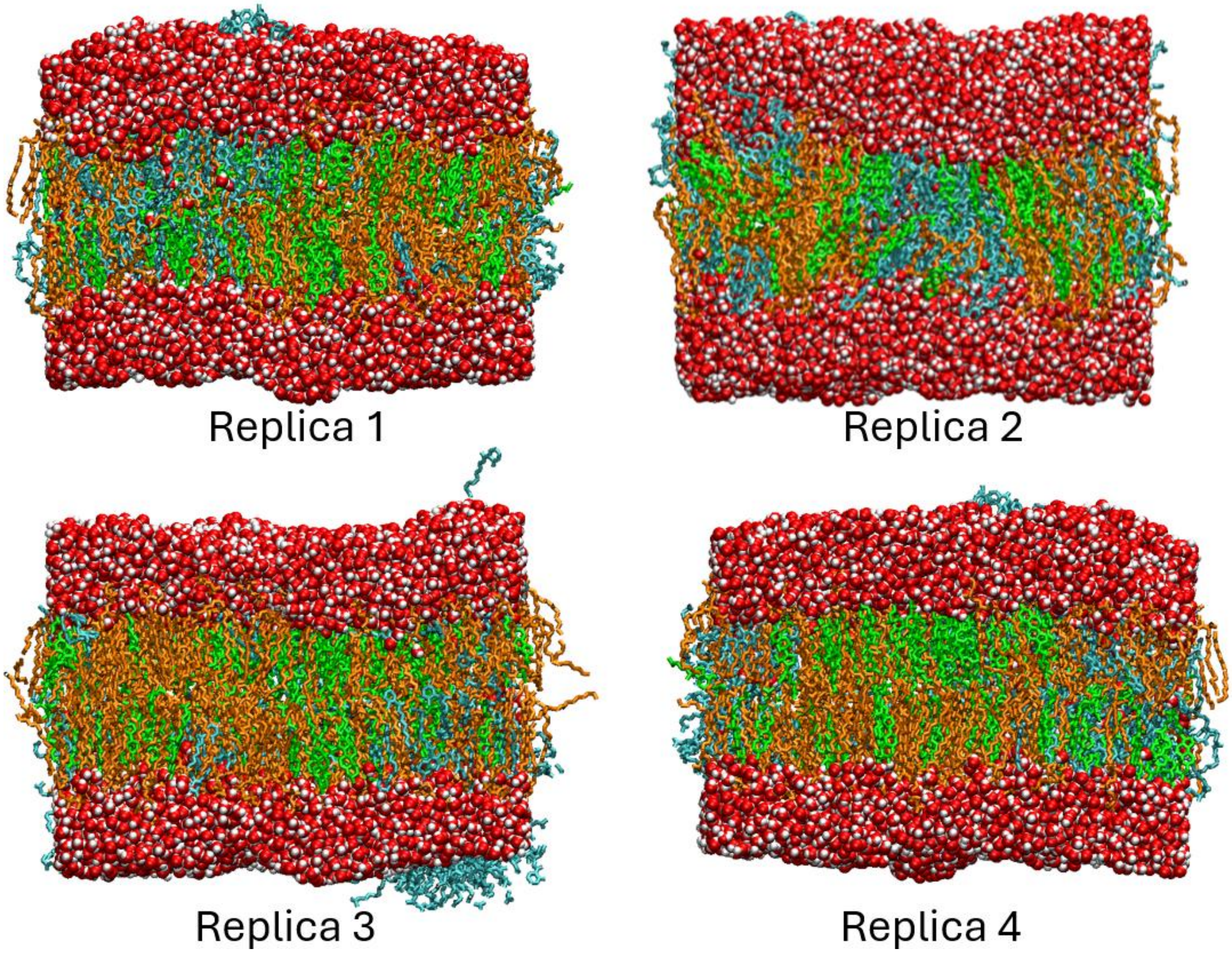

Supplementary Fig. 32 | Membrane Stability After 1 μs of Unbiased Simulation. Snapshots of the last frames for unbiased 1 μs replicas as described in Fig. 6 of the main text. Water molecules within 2.5 nm of the membrane are all shown in space-filling mode to highlight the stability of the membrane after 1 μs of simulation time (peptide omitted for clarity). The relatively few water molecules in the membrane are incorporated into SNAC clusters (cyan), thereby maintaining the membrane's integrity throughout the simulation.

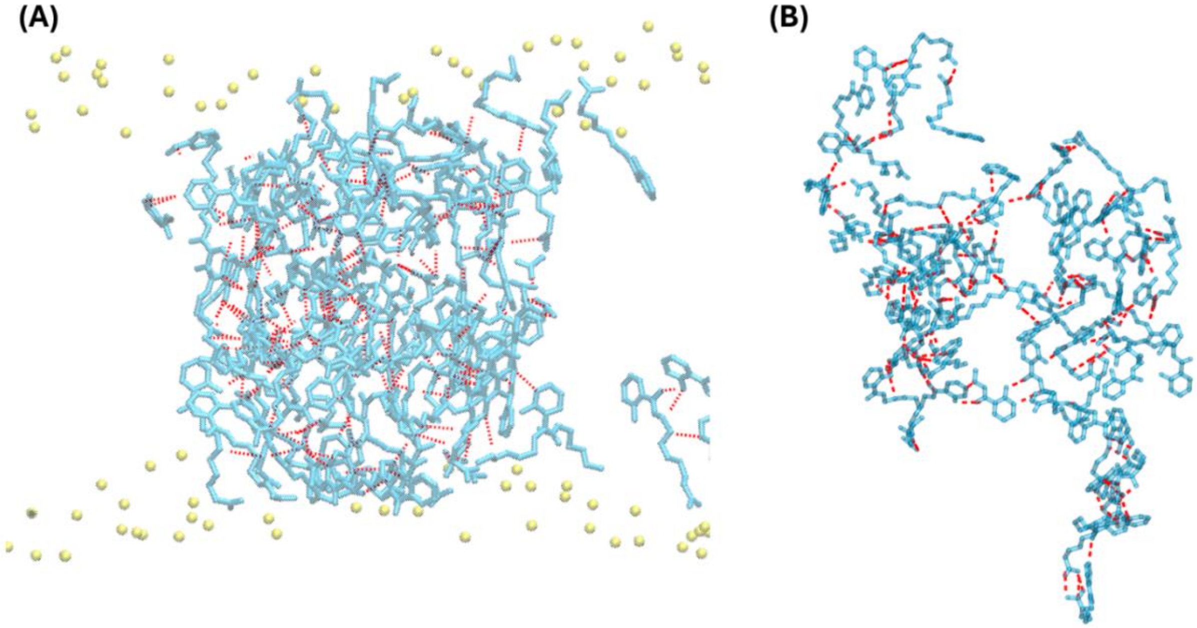

Supplementary Fig. 33 | Hydrogen-bond Driven SNAC Aggregate Formation in Nonpolar Environments. The figure illustrates snapshots of a prototypical branched hydrogen bonding network formed with SNAC inside the membrane **(A)** and from the simulation of SNAC aggregating in CH_2Cl_2 **(B).** Hydrogen bonds are illustrated with dashed red lines.

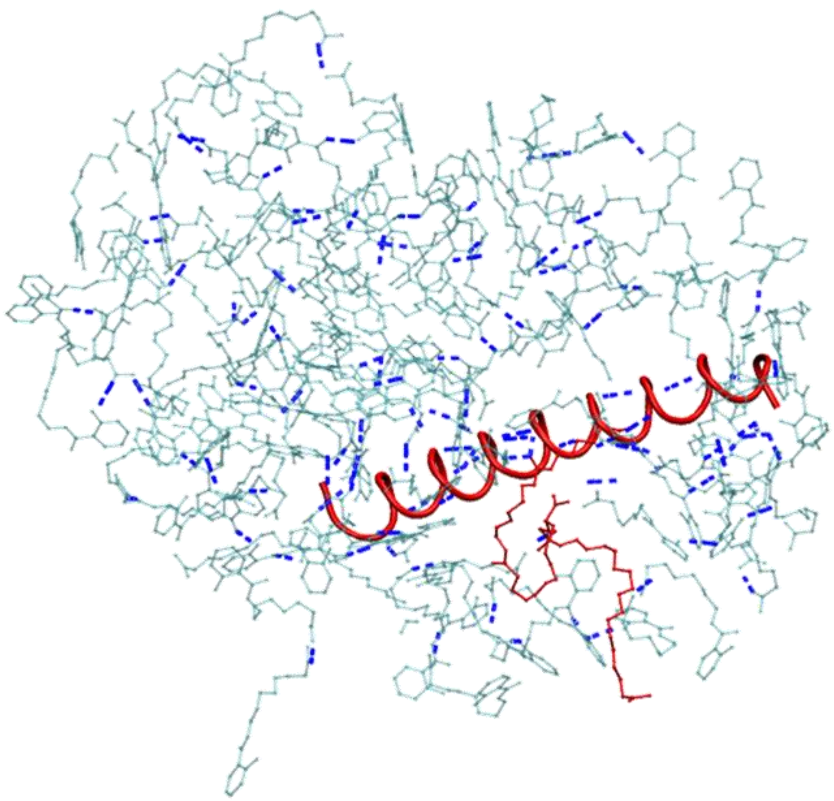

Supplementary Fig. 34 | Hydrogen-bonded Network of SNAC Aggregated with Semaglutide in a Nonpolar Environment. SNAC forms hydrogen bonds (blue dotted lines) with semaglutide to stabilize the peptide in CH_2Cl_2, as also shown in the structure shown in Fig. 5A.

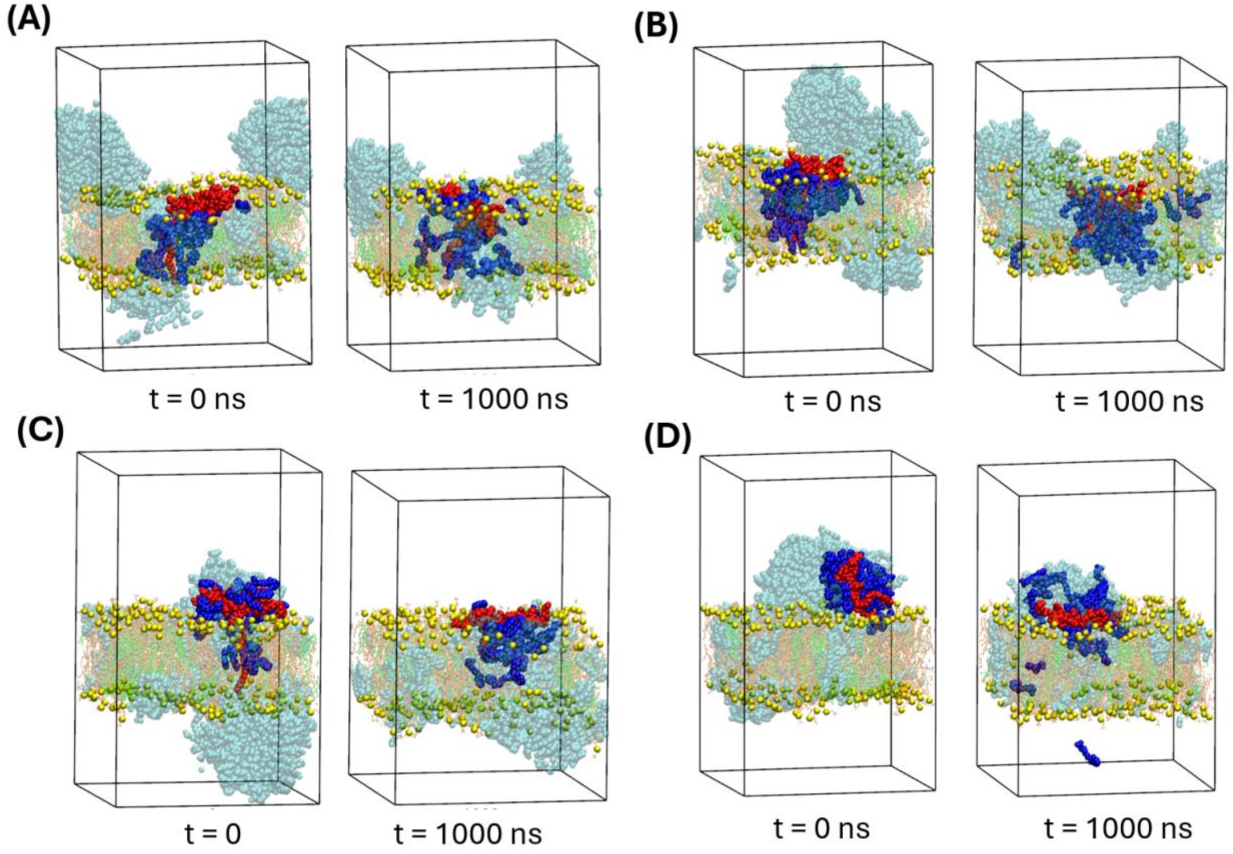

Supplementary Fig. 35. | Snapshots of Unbiased 1-μs Simulations. Initial and last frames of Supplementary Videos 11 (**A**, replica 1), 12 (**B**, replica 2), 13 (**C**, replica 3), and 14 (**D**, replica 4) of unbiased 1-μs simulations of semaglutide (red) slowly beginning to sink into the membrane. All SNAC molecules initially found within 7.5 Å of semaglutide are colored dark blue and exhibit significant movement/rearrangement throughout the simulation. During the simulations, semaglutide started to spontaneously sink into the membrane, and SNAC molecules rearranged dynamically, even though the lipid tail was not pulled as deeply into the membrane as for replicas 3 and 4.

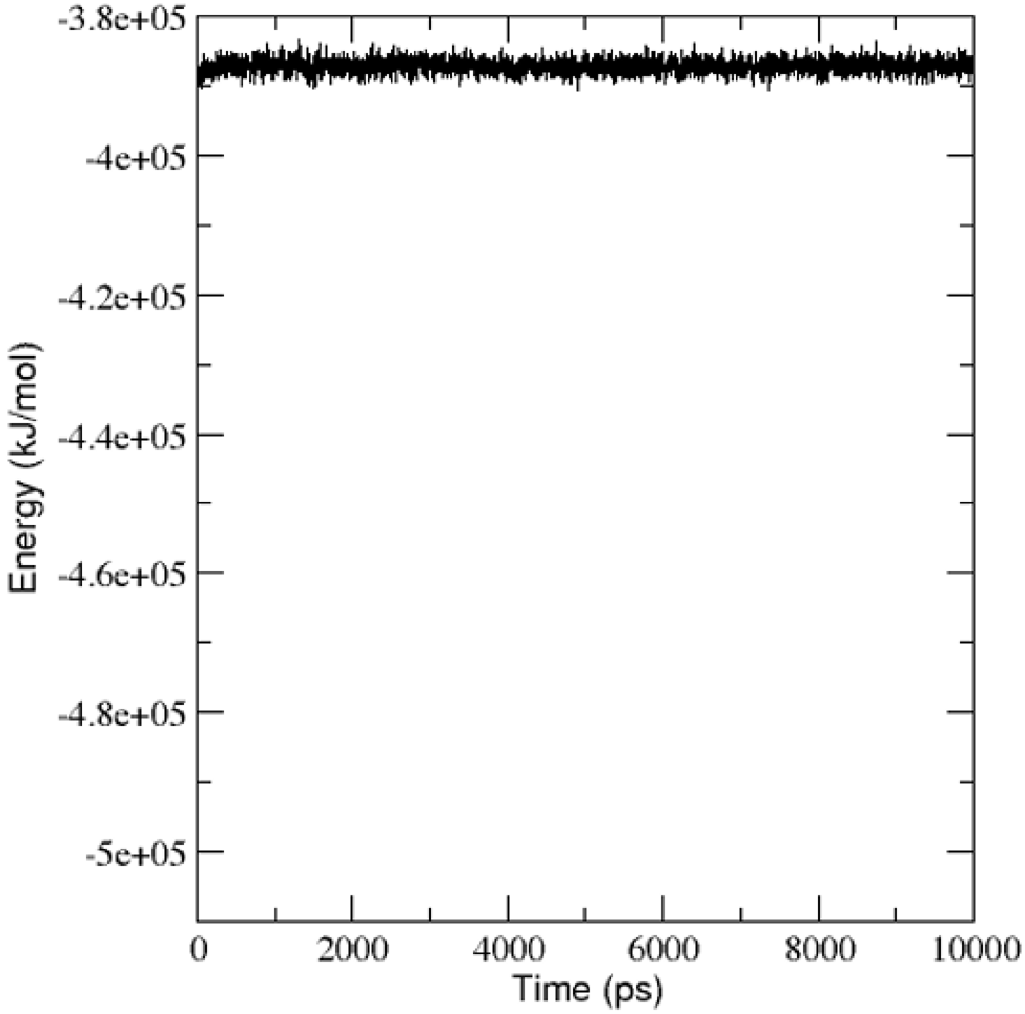

Supplementary Fig. 36 | Total Energy *vs.* Time Plot for the 10-ns CpHMD Equilibration Run Generating the Initial Frame of the Production Run Shown in Supplementary Video 1. The plot shows that the total energy of the system is fully equilibrated after just a few hundred ps. Source data are provided as a Source Data file.

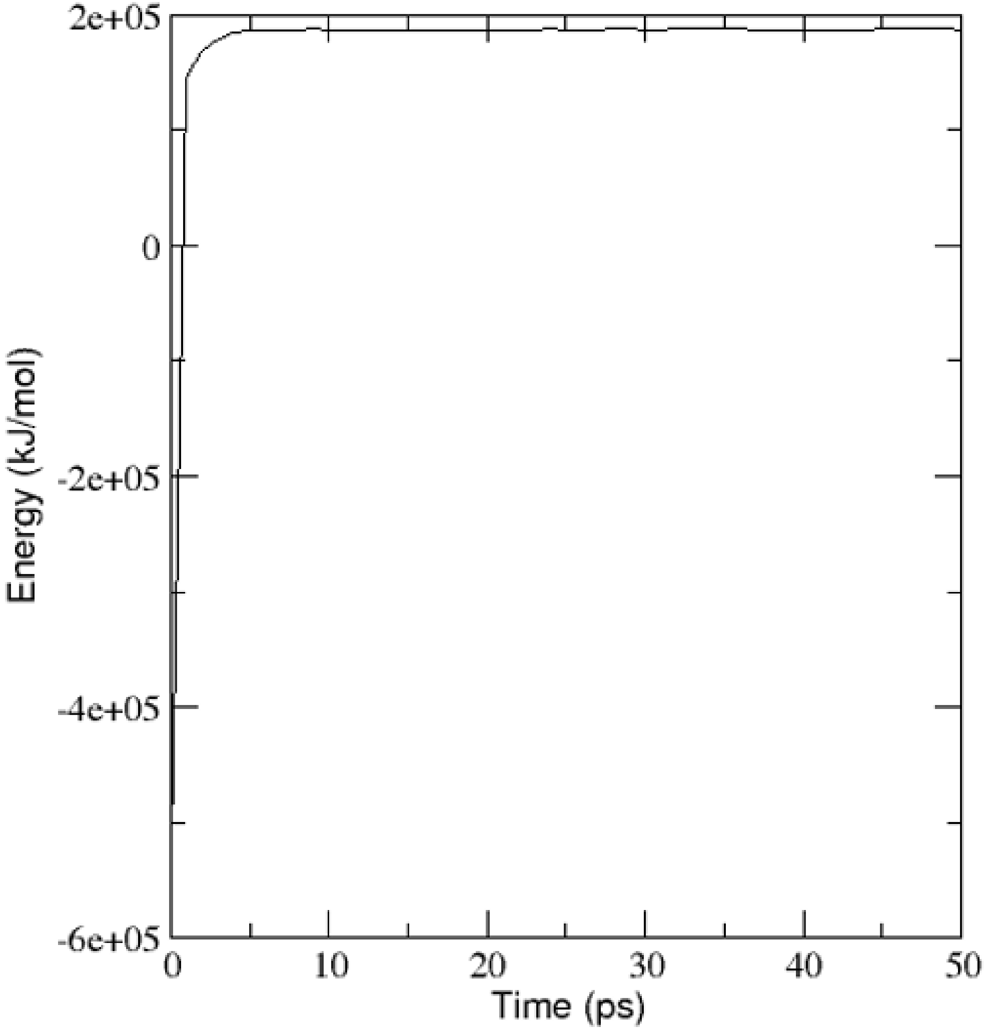

Supplementary Fig. 37 | Total Energy *vs.* Time Plot for the 100-ps CpHMD Equilibration Run Generating the Initial Frame of the Production Run Shown in Supplementary Video 2. The plot shows that the total energy of the system is fully equilibrated after ~20 ps. Source data are provided as a Source Data file.

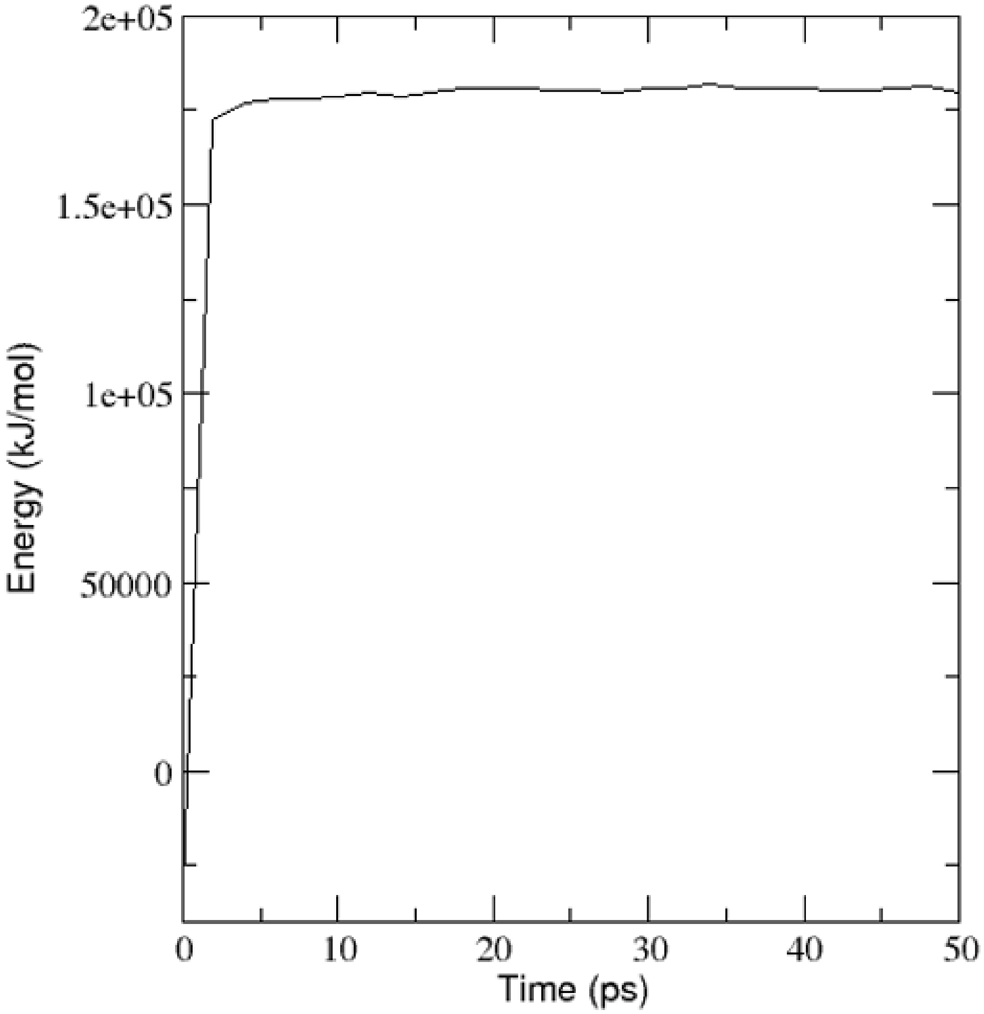

Supplementary Fig. 38 | Total Energy *vs.* Time Plot for the 50-ps CpHMD Equilibration Run Generating the Initial Frame of the Production Run Shown in Supplementary Video 3. The plot shows that the total energy of the system is fully equilibrated after ~10 ps. Source data are provided as a Source Data file.

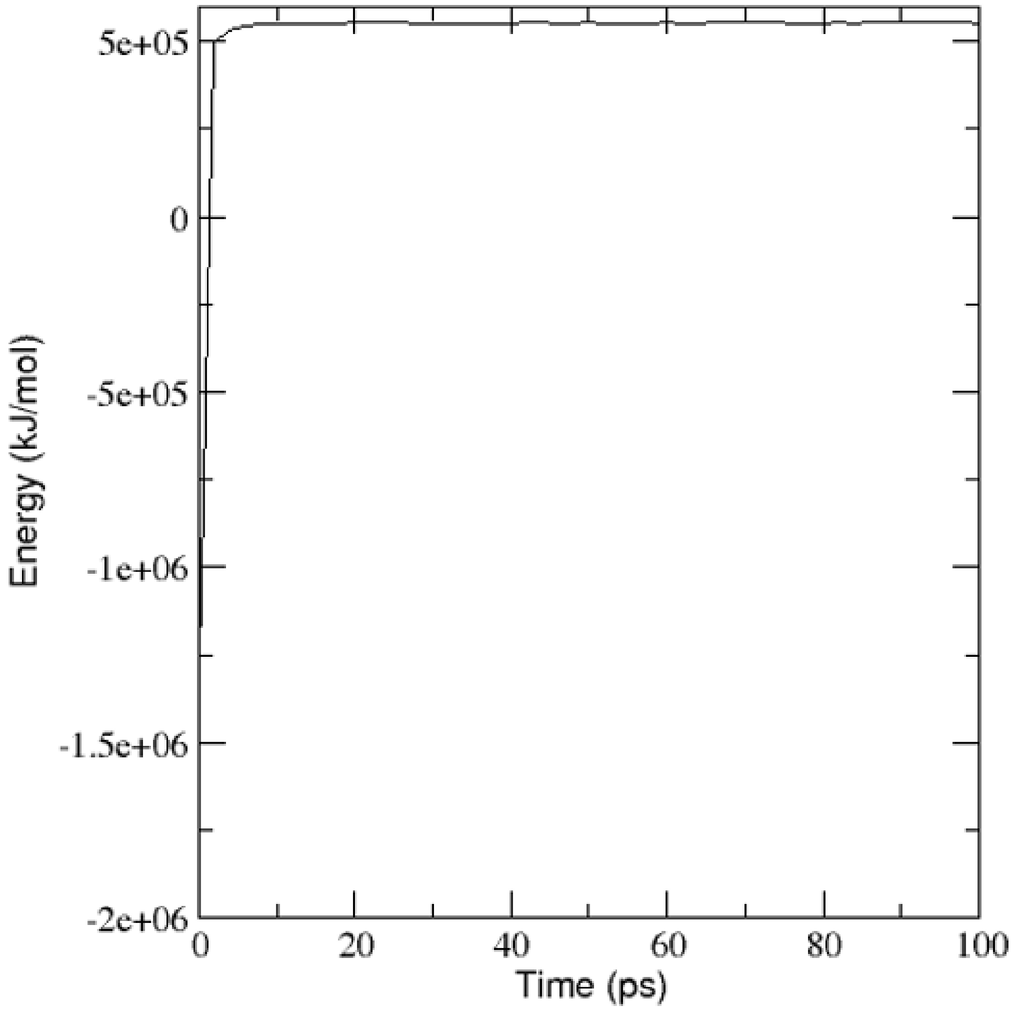

Supplementary Fig. 39 | Total Energy *vs.* Time Plot for the 100-ps CpHMD Equilibration Run Generating the Initial Frame of the Production Run Shown in Supplementary Video 4. The plot shows that the total energy of the system is fully equilibrated after ~20 ps. Source data are provided as a Source Data file.

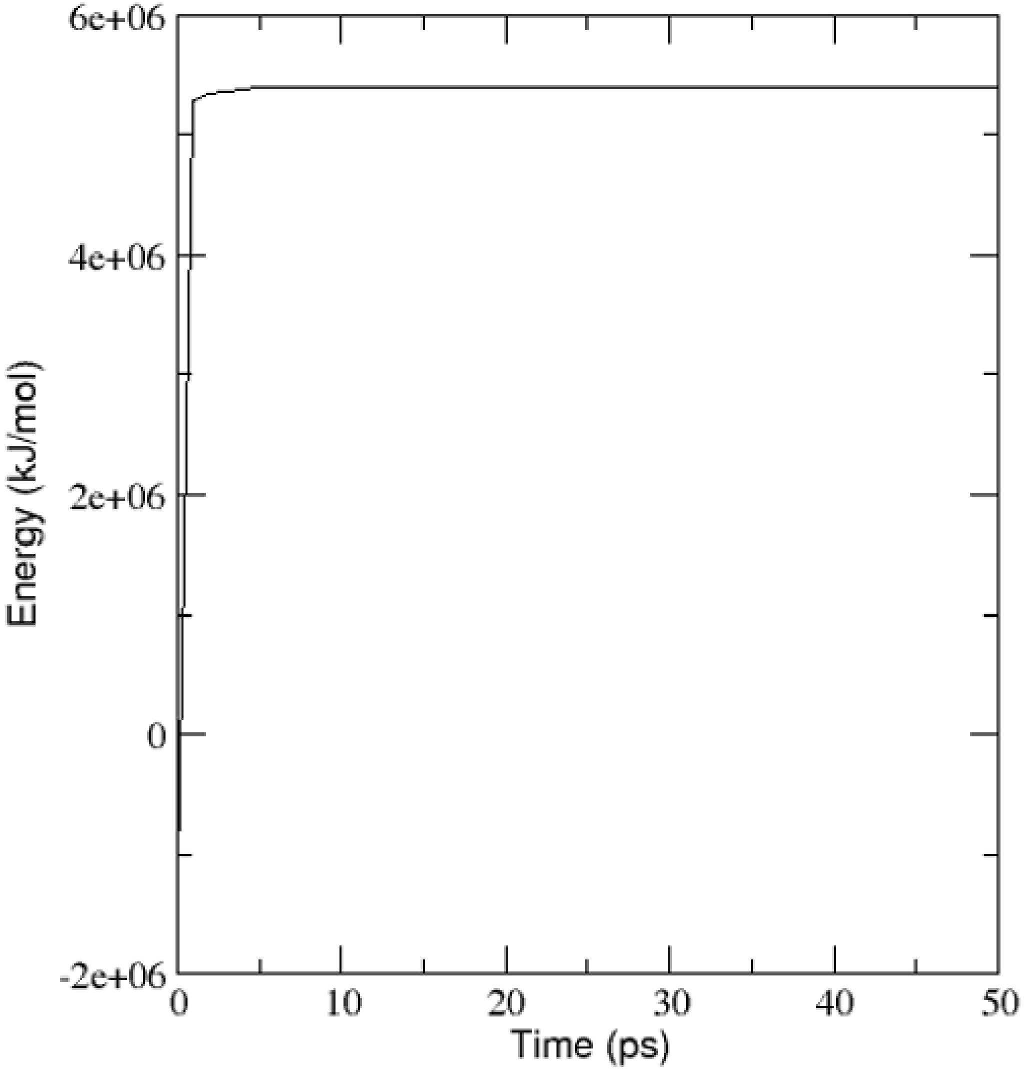

Supplementary Fig. 40 | Total Energy *vs.* Time Plot for the 100-ps CpHMD Equilibration Run Generating the Initial Frame of the Production Run Shown in Supplementary Video 5. The plot shows that the total energy of the system is fully equilibrated after ~20 ps. Source data are provided as a Source Data file.

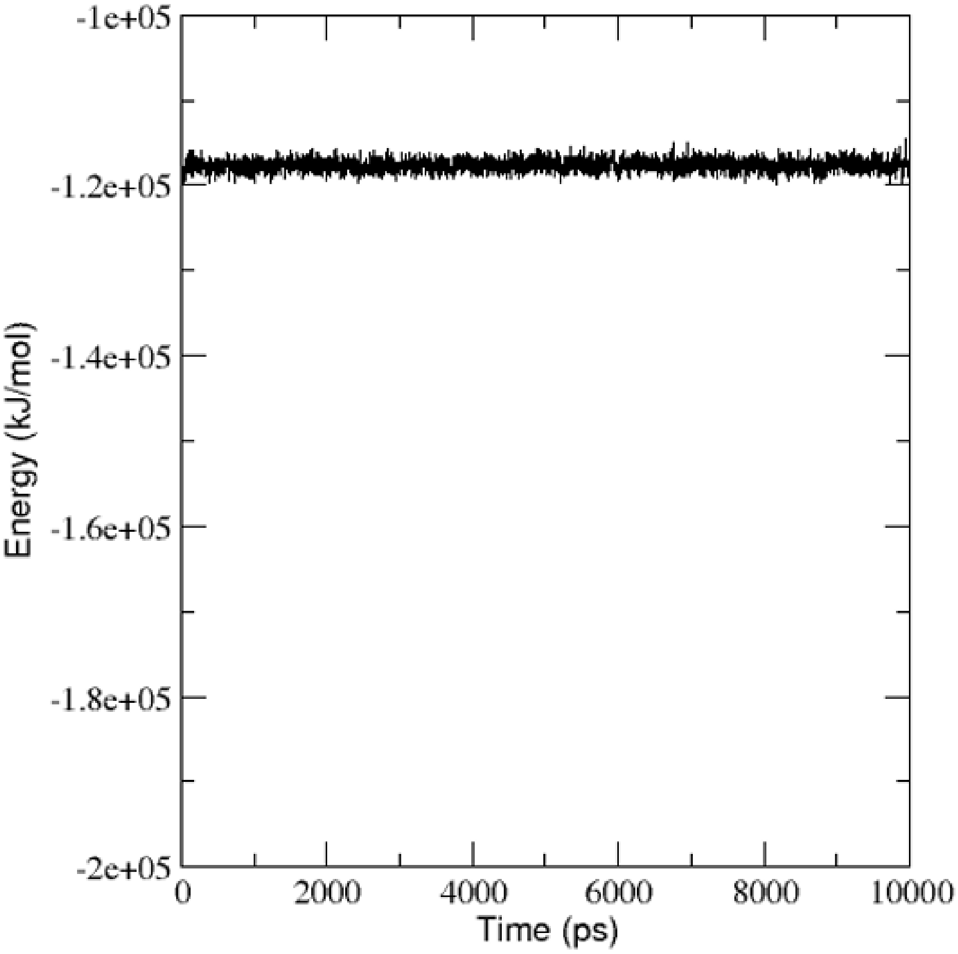

Supplementary Fig. 41 | Total Energy *vs.* Time Plot for the 10-ns CpHMD Equilibration Run Generating the Initial Frame of the Production Run Shown in Supplementary Video 6. The plot shows that the total energy of the system is fully equilibrated after ~1 ns. Source data are provided as a Source Data file.

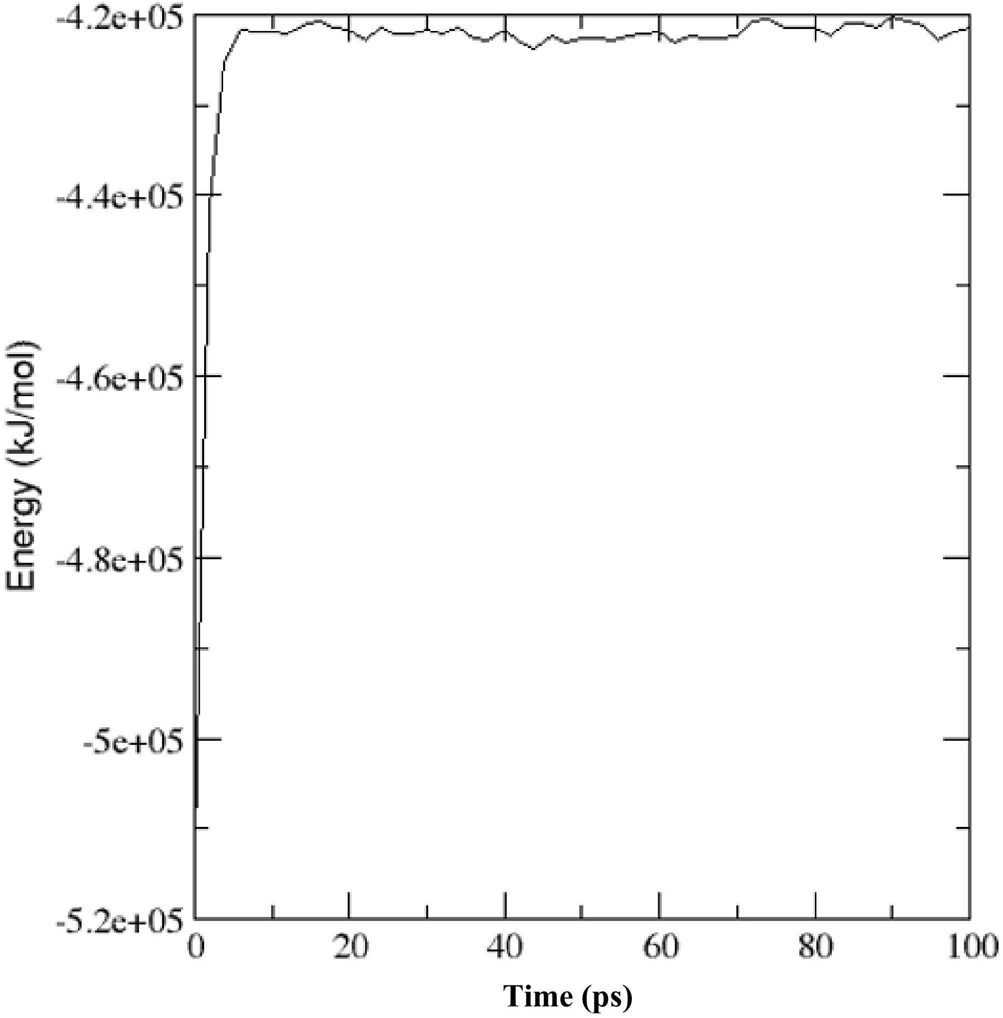

Supplementary Fig. 42 | Total Energy *vs.* Time Plot for the 100 ps CpHMD Equilibration Run Generating the Initial Frame of the Production Run Shown in Supplementary Video 7. The plot shows that the total energy of the system is fully equilibrated after ~10 ps. Source data are provided as a Source Data file.

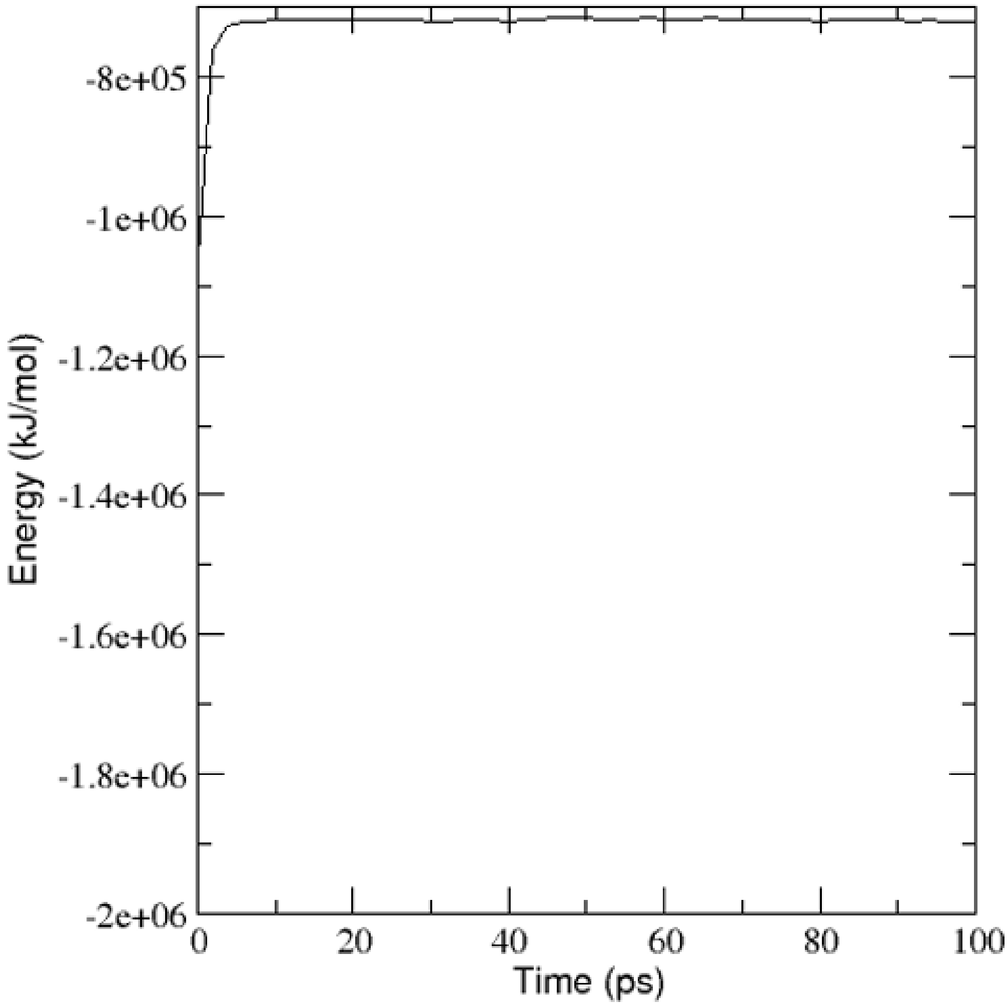

Supplementary Fig. 43 | Total Energy *vs.* Time Plot for the 100 ps CpHMD Equilibration Run Generating the Initial Frame of the Production Run Shown in Supplementary Video 8. The plot shows that the total energy of the system is fully equilibrated after ~10 ps. Source data are provided as a Source Data file.

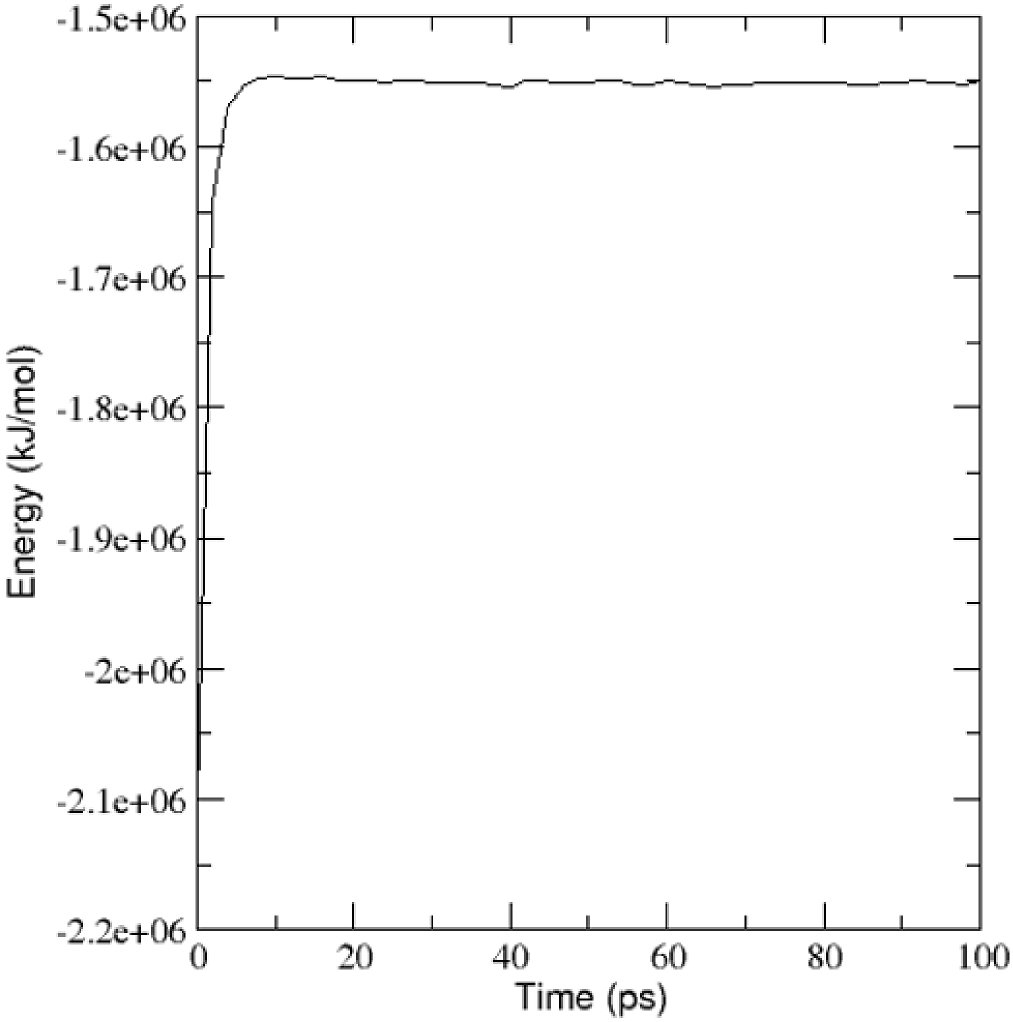

Supplementary Fig. 44 | Total Energy *vs.* Time Plot for the 100 ps CpHMD Equilibration Run Generating the Initial Frame of the Production Run Shown in Supplementary Video 9. The plot shows that the total energy of the system is fully equilibrated after ~10 ps. Source data are provided as a Source Data file.

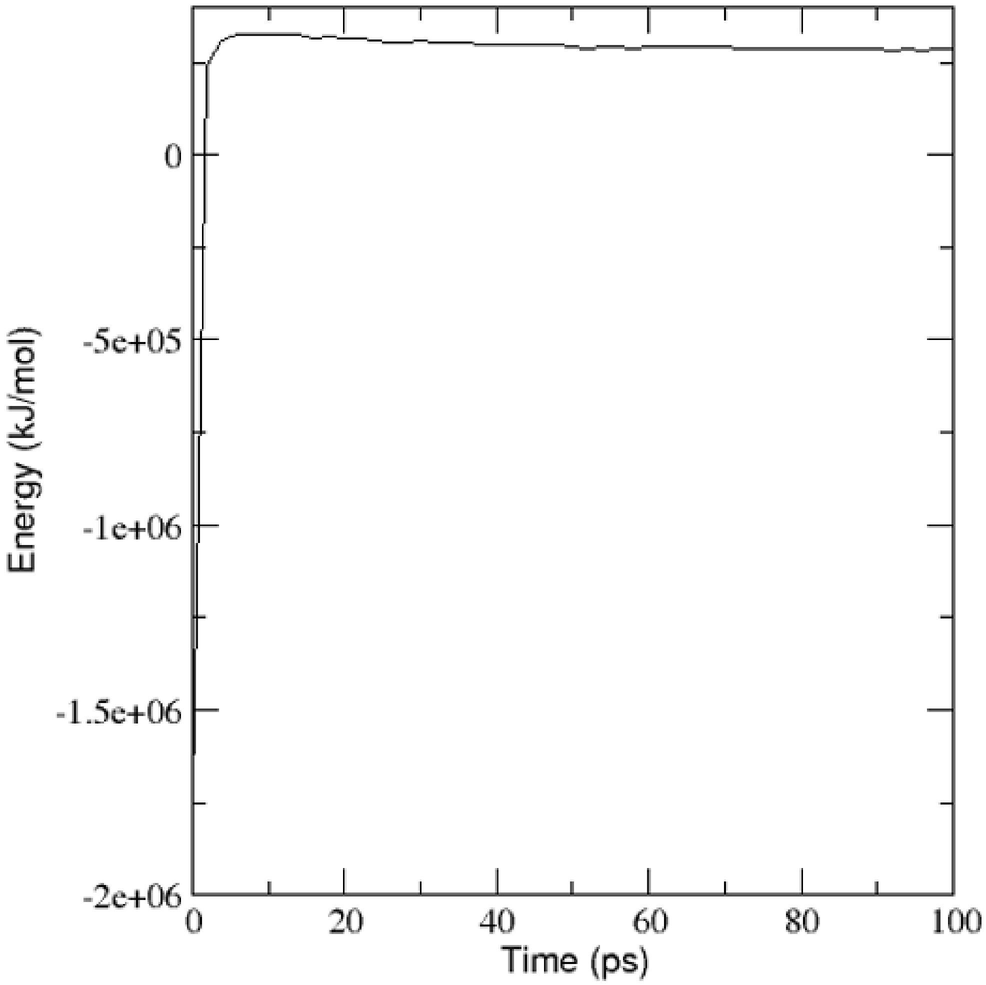

Supplementary Fig. 45 | Total Energy *vs.* Time Plot for the 100-ps CpHMD Equilibration Run for the Self-Assembly Shown in Supplementary Video 10. The plot shows that the total energy of the system is fully equilibrated after ~5 ps. Source data are provided as a Source Data file.

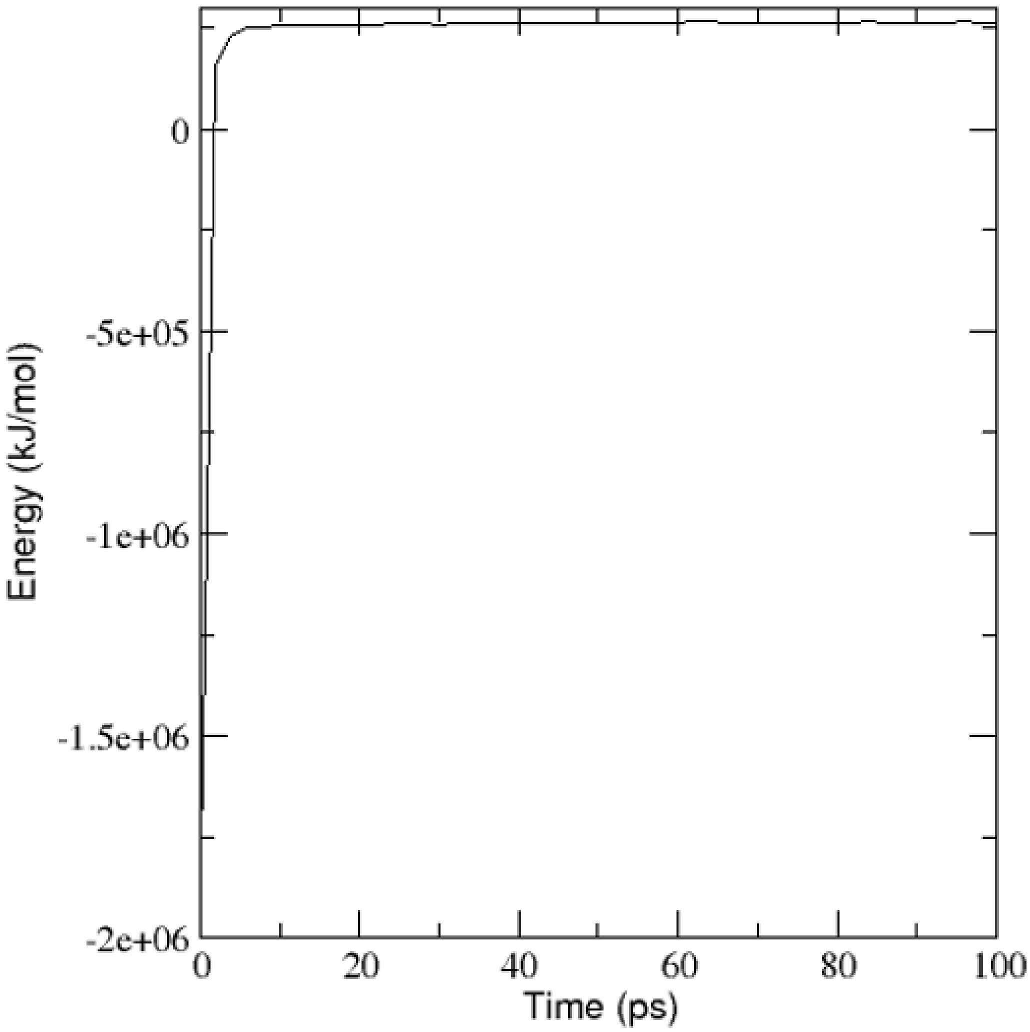

Supplementary Fig. 46 | Total Energy *vs.* Time Plot for the 100-ps CpHMD Equilibration Run Generating the Initial Frame of the Production Run for Replica 1 Shown in Supplementary Video 11. The plot shows that the total energy of the system is fully equilibrated after ~5 picoseconds. Source data are provided as a Source Data file.

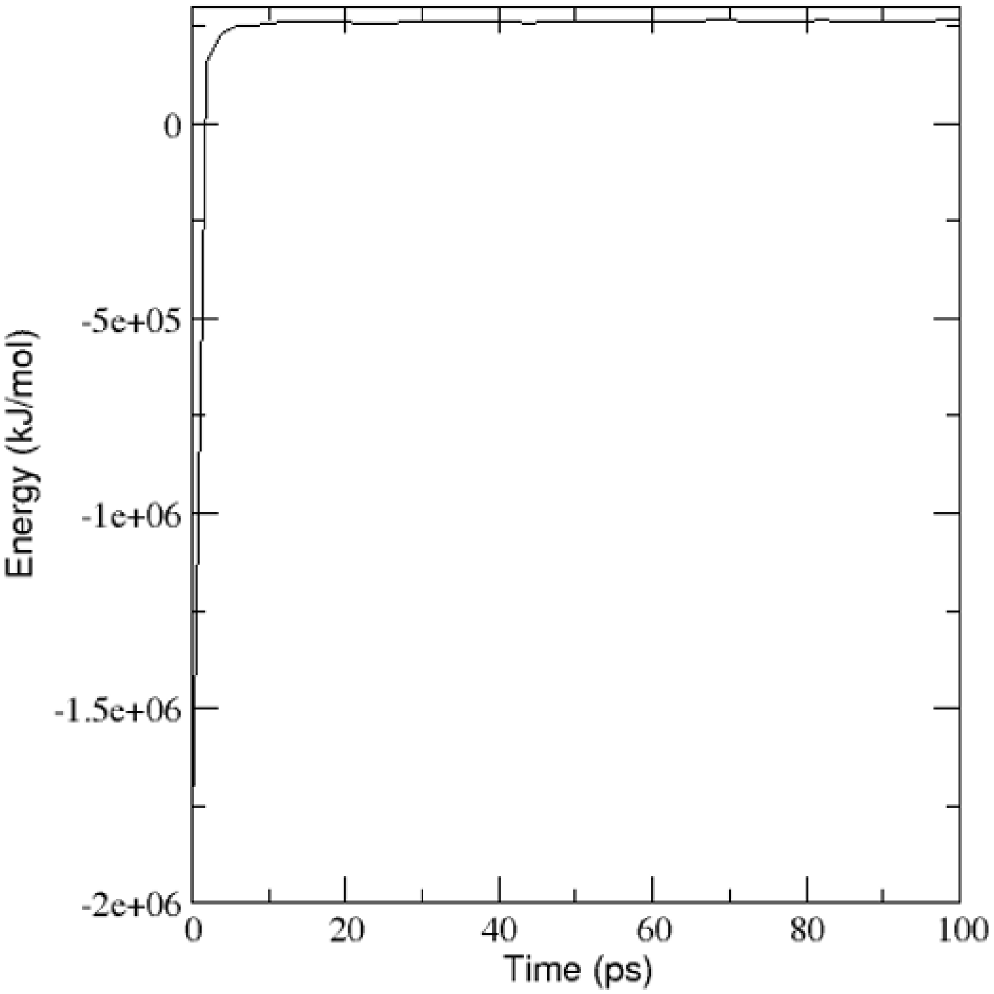

Supplementary Fig. 47 | Total Energy *vs.* Time Plot for the 100-ps CpHMD Equilibration Run Generating the Initial Frame of the Production Run for Replica 2 Shown in Supplementary Video 12. The plot shows that the total energy of the system is fully equilibrated after just ~10 picoseconds. Source data are provided as a Source Data file.

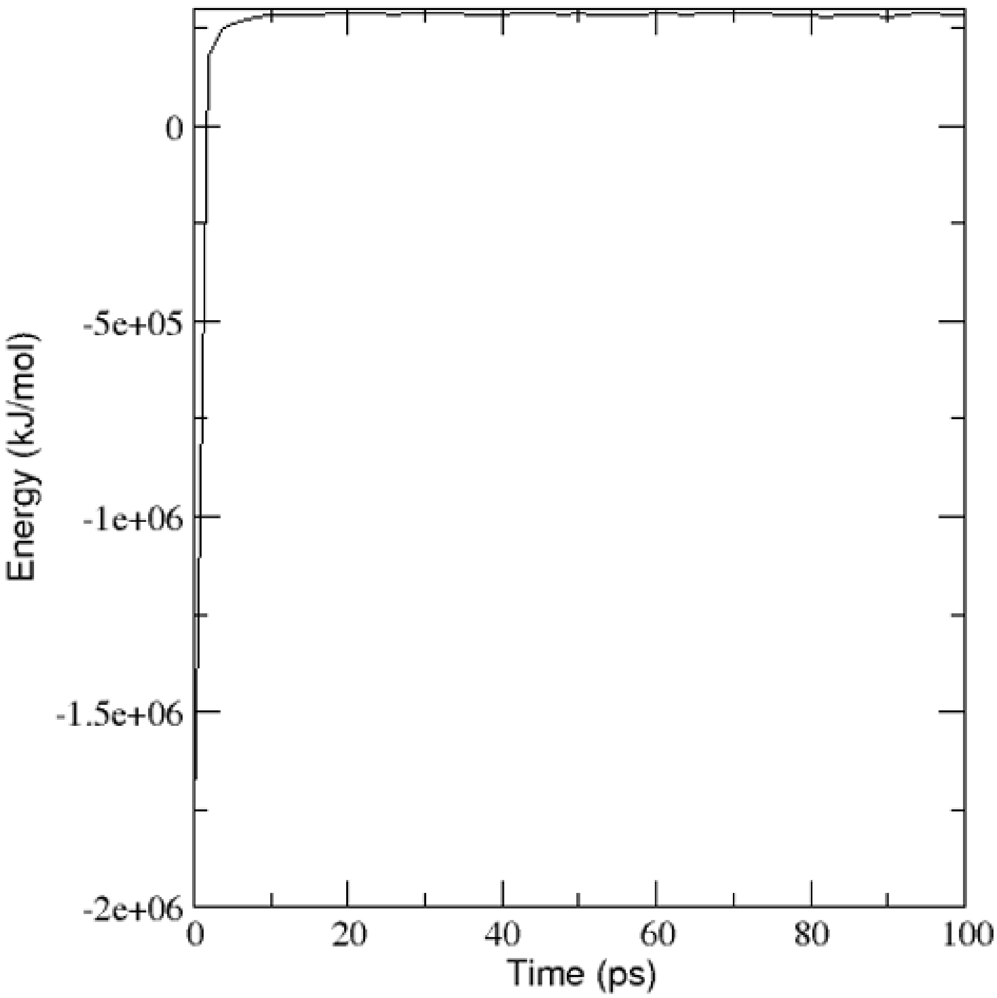

Supplementary Fig. 48 | Total Energy *vs.* Time Plot for the 100-ps CpHMD Equilibration Run Generating the Initial Frame of the Production Run for Replica 3 Shown in Supplementary Video 13. The plot shows that the total energy of the system is fully equilibrated after ~5 ps. Source data are provided as a Source Data file.

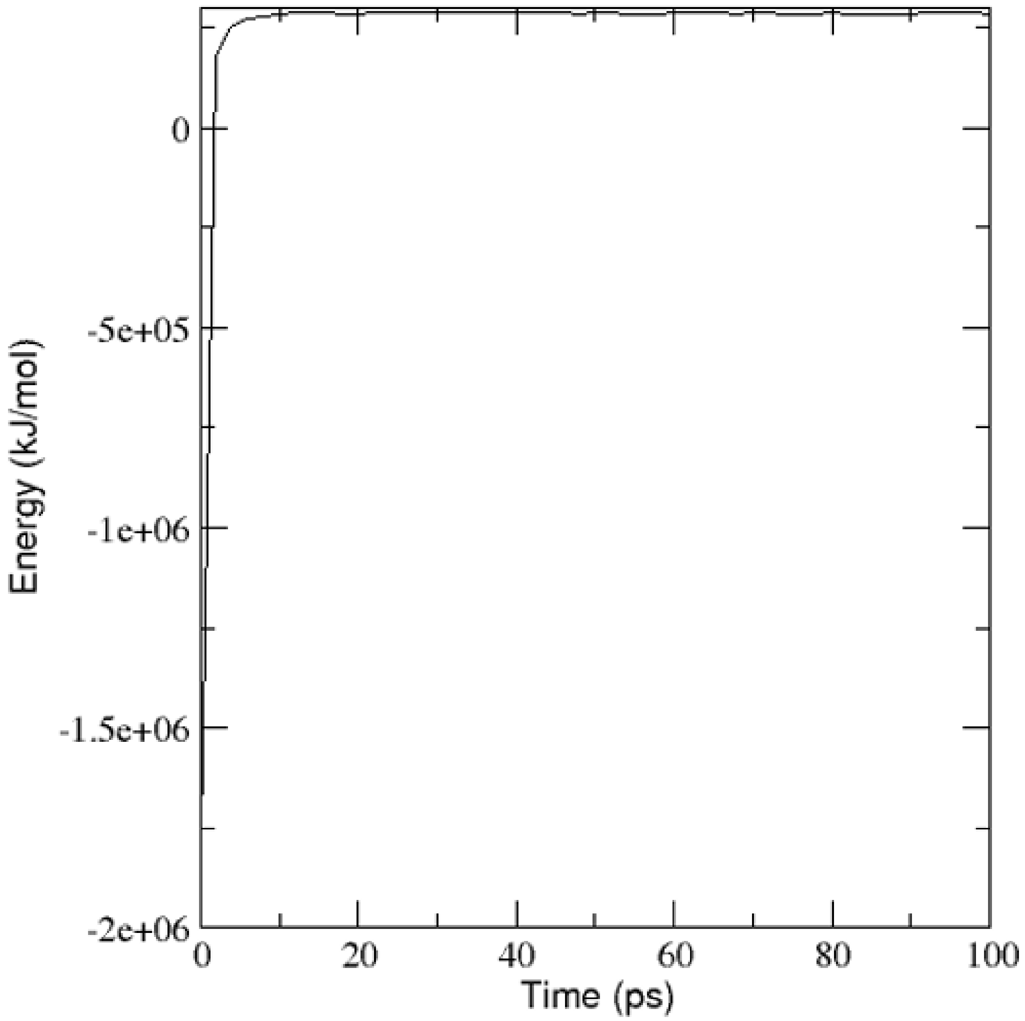

Supplementary Fig. 49 | Total Energy *vs.* Time Plot for the 100-ps CpHMD Equilibration Run Generating the Initial Frame of the Production Run for Replica 4 Shown in Supplementary Video 14. The plot shows that the total energy of the system is fully equilibrated after ~5 ps. Source data are provided as a Source Data file.

SUPPLEMENTARY TABLES

Supplementary Table 1 | Validated parameters for non-standard ionizable residues utilized in the C*p*HMD simulations. Additional modified forcefield parameters used to help increase the conformational sampling of titratable carboxylic acid residues are also provided. Line drawings of parameterized small molecules are provided as insets with corresponding atom labels.

[SNAC]

incl = SNAC SNAH

atoms = C14 C15 O2 O3 H2

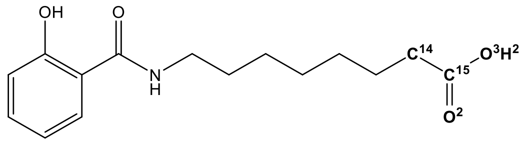

qqA = -0.160 0.727 -0.575 -0.605 0.430

pKa_1 = 5.01

qqb_1 = -0.281 0.620 -0.760 -0.760 0.000

dvdl_1 = -2321 7625 -100030 6405 -2003 3002 -583.9 6.48

[SCH1]

incl = SCH1

atoms = C21 C22 C23 C42 N5 O9 O8 H76 H42 C24

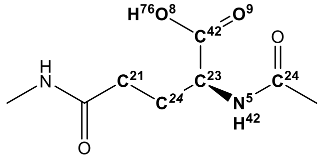

qqA = -0.200 -0.195 0.035 0.809 -0.489 -0.566 -0.521 0.290 0.359

pKa_1 = 3.50

qqb_1 = -0.207 -0.152 -0.184 0.633 -0.461 -0.760 -0.760 0.000 0.431 0.524

dvdl_1 = -88.941 -132.473 769.921 -969.232 525.508 -163.886 -580.806 -73.567

[SCH2]

incl = SCH2

atoms = O5 C41 O6 C40 H75

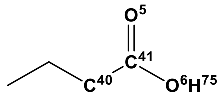

qqA = -0.605 0.727 -0.575 -0.159 0.430

pKa_1 = 4.82

qqb_1 = -0.760 0.620 -0.760 -0.280 0.000

dvdl_1 = -1286.315 3126.335 -2734.448 1145.279 -516.719 203.79 -548.893 46.788

[OLET]

incl = OLE OLEH

atoms = H1 O1 O2 C1 C2

qqA = 0.440 -0.610 -0.550 0.750 -0.210

pKa_1 = 4.8

qqb_1 = 0.000 -0.760 -0.760 0.620 -0.280

dvdl_1 = -43.047 213.875 -336.373 -103.767 397.505 -192.068 -555.38 22.658

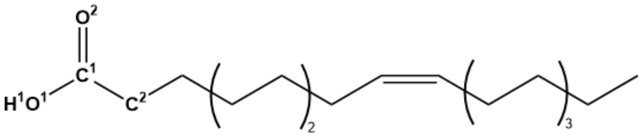

Additional CHARMM36 forcefield parameters for carboxylic acids

i	j	k	l	func	phi0	cp	mult
CG321	CG2O2	OG311	HPG1	9	180.00	-6.0000	2
CG321	CG2O2	OG311	HPG1	9	180.00	1.5000	4

Supplementary Table 2 | Molecular Dynamics Simulation Checklist

**Reliability and reproducibility checklist for molecular dynamics simulations**<br>***All boxes must be marked YES by acceptance unless "Response not needed if No".**	**Yes**	**No**	**Response (Please state where this information can be found in the text)**
**1. Convergence of simulations and analysis**			
1a. Is an evaluation presented in the text to show that the property being measured has equilibrated in the simulations (*e.g.* time-course analysis)?	☒	☐	Time-course analysis (Total Energy *vs.* Time) has been performed for all the simulations during the equilibration runs. The corresponding time-course analysis plots are provided in Supplementary Figs. 36–49. The lengths of the equilibration CpHMD simulations are indicated in the corresponding figure captions.
1b. Then, is it described in the text how simulations are split into equilibration and production runs and how much data were analyzed from production runs?	☒	☐	Details for the theoretical simulations (including model system preparation and equilibration) are provided in the supplementary methods section.
1c. Are there at least 3 simulations per simulation condition with statistical analysis?	☒	☒	In part. The new parameters for ionizable groups were validated with 10 independent replicas (all started from different random seeds), while the umbrella sampling experiments contained dozens of overlapping, distinct sampling windows as detailed in Supplementary Figs. 17, 18, and 24. Finally, the unbiased 1 μs simulation of the peptide interacting with SNAC and the membrane was performed in quadruplicate using different random seeds and also different initial model structures (with the semaglutide tail inserted to varying degrees into the membrane in the initial structure) as detailed in the main text and the Supplementary Information.

1d. Is evidence provided in the text that the simulation results presented are independent of initial configuration?	☒	☐	Systems were either assembled randomly and/or run multiple time with different random seeds. Furthermore, Supplementary Figs. 9, 11, 13, and 15 show the efficient sampling of the lambda-parameter space (in the form of histograms) during the 10 distinct 100-ns simulation runs performed for the validation runs of the CpHMD parameters.
**2. Connection to experiments**			
2a. Are calculations provided that can connect to experiments (*e.g.* loss or gain in function from mutagenesis, binding assays, NMR chemical shifts, J-couplings, SAXS curves, interaction distances or FRET distances, structure factors, diffusion coefficients, bulk modulus and other mechanical properties, *etc.*)?	☒	☐	Theoretical simulations are support by NMR (chemical shifts, diffusion coefficients, and NOESY cross peak intensities) as well as DLS spectra. These experimental results are shown in Table 1, Fig. 4, and Supplementary Figs. 1–4, 28, and 29.
**3. Method choice**			
3a. Do simulations contain membranes, membrane proteins, intrinsically disordered proteins, glycans, nucleic acids, polymers, or cryptic ligand binding?	☒	☐	The compositions for model membranes are justified based on prior literature, which is cited in the main text. Furthermore, for the amount of SNAC present in our simulations, our simulations used 400 SNACs per semaglutide molecule in the simulation box, which accurately reflects the experimental ratio of permeation enhancer to peptide drug. Furthermore, it is generally believed that locally, i.e., directly around the tablet, the SNAC concentration is increased significantly compared to what would be expected after even distribution in the stomach, since the tablet is sitting directly on the stomach epithelia (as described by Dr. Brayden's work: See: (Brayden *et al.*, *Pharmaceutics* **2019**,

			11, 78, Ref. 39 in our manuscript). Specifically, a prior study by Novo Nordisk (see: Knudsen *et al.*, *Sci. Transl. Med.* **2018**, 10, *467*, Ref. 5 in our manuscript) measured the SNAC concentration directly underneath the tablet and found the local SNAC concentration to be ~280 mM, which is reasonably well represented by our simulations, in which the overall SNAC concentration was ~350 mM.<br><br>For the solution pH (5.0) employed in our simulations, we also oriented our study based on the previous literature. Specifically, the prior literature has established that the high local SNAC concentration in the direct vicinity of the tablet acts as a buffer, and effectively raises the local solution pH in the stomach to a degree, which mostly inactivates pepsin and protects the therapeutic peptide from being rapidly degraded by pepsin in the stomach. For a recent review on this topic, see: Brayden *et al.*, *Pharmaceutics* **2019**, 11, 78, (Ref. 39 in our revised manuscript). Based on the currently available literature, this inactivation of pepsin occurs generally at or slightly above a pH of 5. (See, for example: Sleisenger and Fordtran's Gastrointestinal and Liver Disease (Ninth Edition), Volume 1, 2010, Pages 817–832.e7, Chapter 49: Gastric Secretion, Ref. 20 in our revised manuscript),

			which makes a pH of 5 a reasonable bulk-park estimate to simulate gastric absorption in the presence of SNAC.
3b. Is it described in the text whether the accuracy of the chosen model(s) is sufficient to address the question(s) under investigation (e.g. all-atom vs. coarse-grained models, fixed charge vs. polarizable force fields, implicit vs. explicit solvent or membrane, force field and water model, etc.)?	☒	☐	The improved accuracy afforded by using constant pH molecular dynamics methods is described in the main text.
3c. Is the timescale of the event(s) under investigation beyond the brute-force MD simulation timescale in this study that enhanced sampling methods are needed?	☒	☒	In part. (a) Aggregation of peptide, permeation enhancer, and membrane, as well as insertion of the peptide into the membrane are shown to occur within several hundred nanoseconds, which lies within the timescale of the unbiased simulations. (b) Obtaining accurate free energies of membrane insertion required umbrella sampling followed by WHAM analysis, which we performed based on standard best practices described in the literature, as detailed in our manuscript.
If **YES**, are the parameters and convergence criteria for the enhanced sampling method clearly stated?	☒	☐	The restraint collective variables for the umbrella sampling, the force constants used for the umbrella sampling, the convergence criteria used for the WHAM analyses, as well as the methodology used to calculate error bars of resulting PMFs are clearly defined in the corresponding main text figure captions, the Supplementary Methods section, as well as the captions of the relevant Supplementary Figures.
If **NO,** is the evidence provided in the text?	☒	☐	Supplementary Movies 11–14 indicate that the timescale for assembly of the membrane/SNAC/semaglutide system lies in the range of several hundred nanoseconds, which is accessible with the

				scalable CpHMD simulations in GROMACS.
**4. Code and reproducibility**				
4a. Is a table provided describing the system setup that includes simulation box dimensions, total number of atoms, total number of water molecules, salt concentration, lipid composition (number of molecules and type)?		☐	☒	Details of the system setup are included throughout the text, as well as in figure and movie captions.
4b. Is it described in the text what simulation and analysis software and which versions are used?		☒	☐	We used a custom fork of GROMACS 2021 for our CpHMD simulations. The code has been deposited on Github by the groups of Dr. Gerrit Groenhof and Dr. Berk Hess. This is clearly stated in the Methods and Supplementary Methods sections of the paper.
4c. Are other parameters for the system setup described in the text, such as protonation state, type of structural restraints if applied, nonbonded cutoff, thermostat and barostat, etc.?		☒	☐	This information is provided in the Supplementary Methods section.
4d. Are initial coordinate and simulation input files and a coordinate file of the final output provided as supplementary files or in a public repository?		☒	☐	The first and final frames for each simulation are provided in a zipped folder (in .pdb format) provided as Supplementary Data Files.
4e. Is there custom code or custom force field parameters?		☒	☐	Response not needed if No
	If **YES**, are they provided as supplementary files or in a public repository?	☒	☐	Modified force field parameters (for new ionizable groups) are included in Supplementary Table 1.

SUPPLEMENTARY REFERENCES

(1) Aho, N.; Buslaev, P.; Jansen, A.; Bauer, P.; Groenhof, G.; Hess, B. Scalable constant pH molecular dynamics in GROMACS. *J. Chem. Theory Comput.* **2022**, *18*, 6148–6160. DOI: 10.1021/acs.jctc.2c00516.

(2) Huang, J.; Rauscher, S.; Nawrocki, G.; Ran, T.; Feig, M.; de Groot, B. L.; Grubmuller, H.; MacKerell, A. D., Jr. CHARMM36m: An improved force field for folded and intrinsically disordered proteins. *Nat. Methods* **2017**, *14*, 71–73. DOI: 10.1038/nmeth.4067.

(3) Vanommeslaeghe, K.; Hatcher, E.; Acharya, C.; Kundu, S.; Zhong, S.; Shim, J.; Darian, E.; Guvench, O.; Lopes, P.; Vorobyov, I.; *et al.* CHARMM General Force Field: A force field for drug-like molecules compatible with the CHARMM all-atom additive biological force fields. *J. Comput. Chem.* **2010**, *31*, 671–690. DOI: 10.1002/jcc.21367.

(4) Jorgensen, W. L.; Chandrasekhar, J.; Madura, J. D.; Impey, R. W.; Klein, M. L. Comparison of simple potential functions for simulating liquid water. *J. Chem. Phys.* **1983**, *79*, 926. DOI: 10.1063/1.445869.

(5) Buslaev, P.; Aho, N.; Jansen, A.; Bauer, P.; Hess, B.; Groenhof, G. Best Practices in Constant pH MD Simulations: Accuracy and Sampling. *J. Chem. Theory Comput.* **2022**, *18*, 6134–6147. DOI: 10.1021/acs.jctc.2c00517.

(6) Lee, J.; Cheng, X.; Swails, J. M.; Yeom, M. S.; Eastman, P. K.; Lemkul, J. A.; Wei, S.; Buckner, J.; Jeong, J. C.; Qi, Y.; *et al.* CHARMM-GUI Input generator for NAMD, GROMACS, AMBER, OpenMM, and CHARMM/OpenMM simulations using the CHARMM36 additive force field. *J. Chem. Theory Comput.* **2016**, *12*, 405–413. DOI: 10.1021/acs.jctc.5b00935.

(7) Martinez, L.; Andrade, R.; Birgin, E. G.; Martinez, J. M. PACKMOL: A package for building initial configurations for molecular dynamics simulations. *J. Comput. Chem.* **2009**, *30*, 2157–2164. DOI: 10.1002/jcc.21224.

(8) Essmann, U.; Perera, L.; Berkowitz, M. L.; Darden, T.; Lee, H.; Pedersen, L. G. A smooth particle mesh Ewald method. *J. Chem. Phys.* **1995**, *103*, 8577. DOI: 10.1063/1.470117.

(9) Hess, B.; Bekker, H.; Berendsen, H. J. C.; Fraaije, J. G. E. M. LINCS: a linear constraint solver for molecular simulations. *J. Comput. Chem.* **1997**, *18*, 1463–1472. DOI: 10.1002/(sici)1096-987x(199709)18:12<1463::aid-jcc4>3.0.co;2-h.

(10) Jansen, A.; Aho, N.; Groenhof, G.; Buslaev, P.; Hess, B. Phbuilder: A tool for efficiently setting up constant pH molecular dynamics simulations in GROMACS. *J. Chem. Inf. Model.* **2024**, *64*, 567–574. DOI: 10.1021/acs.jcim.3c01313.

(11) Jansen, A. *https://gitlab.com/gromacs-constantph/phbuilder*. 2023. (accessed May 2023).

(12) Grossfield, A. Grossfield, Alan, "WHAM: the weighted histogram analysis method", version 2.0.11, http://membrane.urmc.rochester.edu/wordpress/?page_id=126.
