# Supplementary material for: Permeation enhancer-induced membrane defects assist the oral absorption of peptide drugs
Source: Nat Commun. 2025 Oct 28;16:9512. doi: 10.1038/s41467-025-64891-0 (PMC12568990; doi:10.1038/s41467-025-64891-0)
Supplement: Supplementary file 2 — Description of Additional Supplementary Files [file 41467_2025_64891_MOESM2_ESM.docx]

**CAPTIONS OF SUPPLEMENTARY VIDEOS**

**Supplementary Video 1 | Trajectory of SNAC Being Pulled Through a Membrane.** The C*p*HMD trajectory shows a pulling simulation of SNAC through a POPC membrane (64 lipids in each leaflet with 0.15 M NaCl, 310.15 K) at *p*H = 5.0. SNAC is shown in space-filling mode. Phospholipid head groups are in yellow. SNAC is shown in cyan, regardless of its protonation state.

**Supplementary Video 2 | Unbiased Trajectory of SNAC Aggregation in Water.** The 50-ns C*p*HMD trajectory shows 50 SNAC molecules (cyan) aggregating in water (0.15 M NaCl, 310.15 K) at *p*H 5. All SNACs are shown in cyan, regardless of their protonation state.

**Supplementary Video 3 | Unbiased Trajectory of SNAC Aggregation in a Nonpolar Solvent.** The 50-ns C*p*HMD trajectory shows 50 SNAC molecules (cyan) aggregating in CH_2_Cl_2_. All SNACs are shown in cyan, regardless of their protonation state.

**Supplementary Video 4 | Unbiased Trajectory of SNAC Aggregation with Semaglutide in a Nonpolar Solvent.** The 100-ns C*p*HMD trajectory shows 50 SNAC molecules (cyan) aggregating with semaglutide (red) in CH_2_Cl_2_. All SNACs are shown in cyan, regardless of their protonation state.

**Supplementary Video 5 | Unbiased Trajectory of SNAC Aggregation with Semaglutide in Water.** The 100-ns C*p*HMD trajectory shows 50 SNAC molecules (cyan) aggregating with semaglutide (red) in water (0.15 M NaCl, 310.15 K) at *p*H 5. All SNACs are shown in cyan, regardless of their protonation state.

**Supplementary Video 6 | Trajectory of Semaglutide’s Lipid Tail Entering a Membrane.** The C*p*HMD trajectory shows a 100-ns pulling simulation of the terminal carboxylic acid function (highlighted in red) of semaglutide’s lipid tail (**S1**) into a POPC lipid bilayer membrane (64 lipids in each leaflet with 0.15 M NaCl, 310.15 K) at *p*H = 5. See Supplementary Fig. 21 for the structure of **S1**. Phospholipid head groups are shown in yellow and the tails in orange.

**Supplementary Video 7 | Self-Assembly of a CTAB Micelle.** The trajectory shows a 100 ns self-assembly of 25 CTAB molecules (100 mM) spontaneously aggregating to form a micelle.

**Supplementary Video 8 | Unbiased Trajectory of SNAC Interactions with a CTAB Micelle.**  The micelle structure from the final frame of Supplementary Video 7 was used to probe the interaction between SNAC molecules and detergent micelles. SNAC (20 molecules, 40 mM, blue) were randomly placed around the CTAB (25 molecules, 50 mM, red) micelle and simulated with C*p*HMD for 100 ns (*p*H = 5.6, 310 K). SNAC molecules quickly aggregate with the CTAB micelle in agreement with NMR and DLS data.

**Supplementary Video 9 | Unbiased Trajectory of CTAB/SNAC Micelles.** The CTAB/SNAC micelle structure was incorporated from the last frame of Supplementary Video 8 to create a larger system containing 4 CTAB/SNAC micelles (SNAC: 80 molecules, 80 mM; CTAB: 100 molecules, 100 mM). This system was simulated with C*p*HMD (*p*H = 5.6, 310 K) for 100 ns. The periodic boundary is defined with a black box, and additional images are projected along the x- and y-axes for clarity.

**Supplementary Video 10 | Unbiased Trajectory of Semaglutide and SNAC Interacting with a POPC Membrane.** The trajectory shows a 100-ns C*p*HMD simulation of semaglutide (red, space-filling mode) in the presence of 400 SNACs (cyan, representing the approximate semaglutide:SNAC ratio in a Rybelsus tablet). During the simulation, semaglutide spontaneously lies down on the membrane surface and starts to sink into the membrane, as described in the main text and Fig. 5. All SNACs are shown in cyan, regardless of their protonation state. Phospholipid head groups are shown in yellow (space-filling mode), cholesterol in gray, and phospholipid tails in magenta.

**Supplementary Video 11 | Unbiased Trajectory of Semaglutide Spontaneously Sinking into a POPC Membrane in the Presence of SNAC.** The trajectory shows a 1-μs CpHMD simulation of semaglutide (red, space-filling mode) in the presence of 400 SNAC molecules (shown in cyan). The simulation system was prepared with the semaglutide lipid tail anchored in the membrane as detailed in the main text. During this simulation, semaglutide started to spontaneously sink into the membrane. All SNACs are shown in cyan, regardless of their protonation state. Phospholipid head groups are shown in yellow (space-filling mode), cholesterol in gray, and phospholipid tails in magenta.

**Supplementary Video 12 | Unbiased Trajectory (Replica 2) of Semaglutide Spontaneously Sinking into a POPC Membrane in the Presence of SNAC.** The trajectory shows a 1-μs C*p*HMD simulation of semaglutide (red, space-filling mode) in the presence of 400 SNAC molecules (shown in cyan and dark blue). The simulation system was prepared with the semaglutide lipid tail anchored in the membrane as detailed in the main text. During this simulation, semaglutide started to spontaneously sink into the membrane, and SNAC molecules rearranged dynamically. SNAC molecules within 7.5 Å are highlighted in dark blue to highlight their movement throughout the simulation. Phospholipid head groups are shown in yellow (space-filling mode), cholesterol in green, and phospholipid tails in orange. The periodic boundary is defined by the black box and a projection on the y-axis is included for clarity.

**Supplementary Video 13 | Unbiased Trajectory (Replica 3) of Semaglutide Spontaneously Sinking into a POPC Membrane in the Presence of SNAC.** The trajectory shows a 1-μs C*p*HMD simulation of semaglutide (red, space-filling mode) in the presence of 400 SNAC molecules (shown in cyan and dark blue). The simulation system was built with the semaglutide lipid tail prepared in a different conformation than for replicas 1 and 2. During this simulation, semaglutide started to spontaneously sink into the membrane, and SNAC molecules rearranged dynamically, even though the lipid tail was not pulled as deeply into the membrane as for replicas 1 and 2. SNAC molecules within 7.5 Å are highlighted in dark blue to highlight their movement throughout the simulation. Phospholipid head groups are shown in yellow (space-filling mode), cholesterol in green, and phospholipid tails in orange. The periodic boundary is defined by the black box and a projection on the y-axis is included for clarity.

**Supplementary Video 14 | Unbiased Trajectory (Replica 4) of Semaglutide Spontaneously Sinking into a POPC Membrane in the Presence of SNAC.** The trajectory shows a 1-μs C*p*HMD simulation of semaglutide (red, space-filling mode) in the presence of 400 SNAC molecules (shown in cyan and dark blue). The simulation system was built with the semaglutide lipid tail prepared in a different conformation than for replicas 1 and 2. During this simulation, semaglutide started to spontaneously sink into the membrane, and SNAC molecules rearranged dynamically, even though the lipid tail was not pulled as deeply into the membrane as for replicas 1 and 2. SNAC molecules within 7.5 Å are highlighted in dark blue to highlight their movement throughout the simulation. Phospholipid head groups are shown in yellow (space-filling mode), cholesterol in green, and phospholipid tails in orange. The periodic boundary is defined by the black box and a projection on the y-axis is included for clarity.

**Supplementary Video 15 | Unbiased Trajectory Showing Membrane Defects Forming and Expanding in the Presence of SNAC.** The 1-μs C*p*HMD trajectory from the first seed (Replica 1) shows SNAC-filled defects forming in the phosphate head group layer (top view, phosphate head groups shown in yellow, SNAC shown in cyan regardless of protonation state, and semaglutide shown in red).

**Supplementary Video 16 | Trajectory of a 200-ns Umbrella Sampling Window with SNAC in the Water Layer (z-distance = –4.0 nm).** The aromatic ring (blue) and the carboxylic acid function (red) of SNAC are both shown in space-filling mode to illustrate the efficient sampling observed between both extended and contracted conformations of SNAC in the water layer.

**Supplementary Video 17 | Trajectory of a 200-ns Umbrella Sampling Window with SNAC in the Membrane (z-distance = –1.1 nm).** The aromatic ring (blue) and the carboxylic acid function (red) of SNAC are both shown in space-filling mode to illustrate the efficient sampling observed between both extended and contracted conformations of SNAC in the membrane.

**CAPTIONS OF SUPPLEMENTARY DATASETS**

**Supplementary Dataset 1 | First and last frames of MD trajectories.** All structures are provided in pdb format.
